# Heparan sulfate proteoglycans mediate Aβ-induced oxidative stress and hypercontractility in cultured vascular smooth muscle cells

**DOI:** 10.1186/s13024-016-0073-8

**Published:** 2016-01-22

**Authors:** Matthew R. Reynolds, Itender Singh, Tej D. Azad, Brandon B. Holmes, Phillip B. Verghese, Hans H. Dietrich, Marc Diamond, Guojun Bu, Byung Hee Han, Gregory J. Zipfel

**Affiliations:** Department of Neurological Surgery, Washington University School of Medicine, Hope Center Program on Protein Aggregation and Neurodegeneration, Charles F. and Joanne Knight Alzheimer’s Disease Research Center, Campus Box 8057, 660 South Euclid Avenue, St. Louis, Missouri 63110 USA; Department of Neurology, Washington University School of Medicine, Hope Center Program on Protein Aggregation and Neurodegeneration, Charles F. and Joanne Knight Alzheimer’s Disease Research Center, St. Louis, Missouri USA; Center for Alzheimer’s and Neurodegenerative Diseases, UT Southwestern, Dallas, Texas USA; Department of Neuroscience, Mayo Clinic, Jacksonville, Florida USA; Department of Pharmacology, AT Still University Health Sciences, Kirksville, Missouri USA

**Keywords:** Heparan sulfate proteoglycans, Alzheimer’s disease, Vascular smooth muscle cells, Cerebrovascular dysfunction, Reactive oxygen species, Oxidative stress, Heparinase, Heparin

## Abstract

**Background:**

Substantial evidence suggests that amyloid-β (Aβ) species induce oxidative stress and cerebrovascular (CV) dysfunction in Alzheimer’s disease (AD), potentially contributing to the progressive dementia of this disease. The upstream molecular pathways governing this process, however, are poorly understood. In this report, we examine the role of heparan sulfate proteoglycans (HSPG) in Aβ-induced vascular smooth muscle cell (VSMC) dysfunction in vitro.

**Results:**

Our results demonstrate that pharmacological depletion of HSPG (by enzymatic degradation with active, but not heat-inactivated, heparinase) in primary human cerebral and transformed rat VSMC mitigates Aβ_1-40_- and Aβ_1-42_-induced oxidative stress. This inhibitory effect is specific for HSPG depletion and does not occur with pharmacological depletion of other glycosaminoglycan (GAG) family members. We also found that Aβ_1-40_ (but not Aβ_1-42_) causes a hypercontractile phenotype in transformed rat cerebral VSMC that likely results from a HSPG-mediated augmentation in intracellular Ca^2+^ activity, as both Aβ_1-40_-induced VSMC hypercontractility and increased Ca^2+^ influx are inhibited by pharmacological HSPG depletion. Moreover, chelation of extracellular Ca^2+^ with ethylene glycol tetraacetic acid (EGTA) does not prevent the production of Aβ_1-40_- or Aβ_1-42_-mediated reactive oxygen species (ROS), suggesting that Aβ-induced ROS and VSMC hypercontractility occur through different molecular pathways.

**Conclusions:**

Taken together, our data indicate that HSPG are critical mediators of Aβ-induced oxidative stress and Aβ_1-40_-induced VSMC dysfunction.

**Electronic supplementary material:**

The online version of this article (doi:10.1186/s13024-016-0073-8) contains supplementary material, which is available to authorized users.

## Background

Alzheimer’s disease (AD) is a progressive amnestic dementia characterized by the deposition of Aβ peptides within the brain parenchyma and cerebrovasculature [1]. While the mechanisms underlying the development and progression of AD remain enigmatic, a growing body of evidence indicates that the pathologic effects of Aβ on cerebral vessels likely play a critical role (for review, see Ref. [2]). Specifically, soluble and insoluble forms of Aβ have been shown to impair CV autoregulation [3–6], reduce cerebral blood flow (CBF) [3, 7, 8], and exacerbate ischemic infarction [9–11]—deleterious effects that are thought to contribute to the progressive dementia of AD. Understanding the mechanisms of these Aβ − induced CV deficits is therefore essential to guide development of novel therapies.

Multiple lines of evidence indicate that Aβ − induced CV deficits are mediated by reactive oxygen species (ROS) (for review, see Ref. [12]). For instance, we have shown that application of exogenous, soluble Aβ (Aβ_1-40_ and Aβ_1-42_ monomers) onto isolated mouse cerebral arterioles leads to significant oxidative stress and vasomotor dysfunction, and that anti-ROS strategies markedly improve these CV deficits [13]. Others have shown that exogenous Aβ monomers applied to the pial surface of live mice cause significant oxidative stress and CV dysfunction, both of which can be attenuated via a variety of anti-ROS interventions [14–16]. Young Tg2576 mice having elevated levels of endogenous soluble Aβ species display substantial oxidative stress and CV deficits, both of which can be inhibited by genetically eliminating the catalytic subunit Nox2 of NADPH oxidase (nicotinamide adenine dinucleotide phosphate-oxidase – a major source of ROS in cerebral vessels) [17]. Fibrillar Aβ in the form of cerebral amyloid angiopathy (CAA) has also been shown to promote CV dysfunction via ROS. First, Garcia-Alloza et al. [18] observed that CAA-laden vessels (but not CAA-free vessels) of aged Tg2576 mice develop severe oxidative stress. Second, Park et al. [19] found that aged Tg2576 mice lacking the Nox2 subunit of NADPH oxidase develop less oxidative stress and no CV deficits compared to age-matched control Tg2576 mice. Though the presence of CAA and its effect on vessel function was not specifically examined in this study, the fact that Tg2576 mice were assessed at an age when CAA is expected [20] suggested that NADPH oxidase-derived ROS may also contribute to CAA-induced CV deficits. Third, we recently reported that administration of the NADPH oxidase inhibitor, apocynin, or the free radical scavenger, tempol, to aged Tg2576 mice significantly improves CV dysfunction and does so by decreasing CAA-induced vasomotor impairment as well as reducing CAA formation itself [21]. As such, modulation of ROS and identification of the upstream inducers of Aβ-mediated ROS production will be instrumental for designing novel therapies to prevent Aβ-induced CV dysfunction and the impact these vascular deficits have on AD dementia.

Heparan sulfate proteoglycans (HSPG) are an attractive upstream target of Aβ-induced ROS production and CV dysfunction. HSPG are complex macromolecules involved in diverse biological processes and are ubiquitously present on the cell surface and in the extracellular matrix [22]. Immunohistochemical studies of post-mortem AD brain suggest that HSPG are associated with the hallmark Aβ pathologies and correlate temporally with Aβ deposition [23, 24]. In vitro experiments demonstrate that HSPG promote Aβ aggregation [25], stabilize Aβ fibrils [26], and inhibit Aβ degradation [26]. HSPG bind Aβ with high affinity and promote its intracellular uptake in multiple cell types [27], including human cerebral vascular smooth muscle cells (VSMC) [28]. In addition, pharmacological and/or genetic depletion of HSPG prevents the intracellular uptake of Aβ and resultant deposition both in vitro [29] and in vivo [30]. These findings implicate HSPG as a key contributor to Aβ metabolism and fibrillogenesis and suggest that it may play a key role in ultimately the development of AD. The role of HSPG in Aβ-induced ROS production and CV dysfunction, however, has yet to be examined.

To date, most studies examining the mechanisms of Aβ-induced CV deficits have focused on the deleterious interaction of Aβ on vascular endothelial cells (VEC) (for review see Ref. [31]). Specifically, Aβ has been shown to induce VEC dysfunction, leading to decreased production of nitric oxide (NO; a potent endothelial-derived vasodilator) and vascular hypercontractility [17]. Several studies, however, have shown that VSMC dysfunction also plays a causal role in Aβ-induced CV deficits, as enhanced ATP-induced constriction (an VEC-independent response) was noted in isolated mouse cerebral arterioles exposed to exogenous Aβ monomers [13] and impaired responses to the VEC-independent vasodilator SNAP was documented in young Tg2576 mice having elevated levels of endogenous soluble Aβ (but no CAA) [6, 17]. Moreover, given the location of fibrillar Aβ within the abluminal portion of the tunica media surrounding VSMC in CAA-laden vessels [32, 33], the VSMC architectural changes and frank cell death that occurs in vessels with CAA deposits [6, 34]. The expectation that markedly elevated levels of soluble Aβ species reside in close proximity to CAA deposits within the perivascular space [35], it is likely that VSMC dysfunction plays an even greater role in CAA-induced CV deficits. We therefore focused our experiments on VSMC as the target of Aβ-mediated toxic effects in an effort to elucidate the upstream molecular events leading NADPH oxidase activation, ROS production, and vascular cell dysfunction.

## Methods

### Reagents

Aβ peptides (Aβ_1-40_, Aβ_40-1_, and Aβ_1-42_) were purchased from American Peptide Company (Sunnyvale, CA), reconstituted in purified water, snap frozen over liquid nitrogen and immediately utilized or stored at -80 °C for no longer than two weeks prior to use. Chondroitinase B and AC were purchased from IBEX Technologies Inc. (Montreal, Quebec, Canada). Purified receptor-associated protein (RAP) was a gift from Dr. Bu (Mayo Clinic, FL). All other reagents, including heparinase enzymes (I and III), were purchased from Sigma-Aldrich (St. Louis, MO).

### Antibodies

The 82E1 (anti-Human Aβ mouse monoclonal antibody), HJ2 (anti-human Aβ35–40) and HJ5-1 (an antibody that selectively recognizes soluble, monomeric anti-human Aβ) monoclonal antibodies were a gift from Dr. Holtzman (Washington University in St. Louis, MO).

### Cell culture

Primary human cerebral VSMC were purchased from ScienCell Research Laboratories (Carlsbad, CA) and grown according to the manufacturer’s instructions. To confirm the data obtained from these cells, primary human cerebral VSMC from another source (Cell Biologics, Chicago, IL) were also used. All human VSMC used in the present study were APOE e3/e3 allele. Transformed rat cerebral VSMC were also used, which were kindly provided by Dr. Diglio, Wayne State University, MI) [36, 37]. Rat cerebral VSMC were grown in Dulbecco’s modified eagle’s medium (DMEM) supplemented with 10 % fetal bovine serum (FBS), 100 U/mL penicillin, and 100 μg/mL streptomycin sulfate. Rat VSMC cell line was established from long-term serial cultures of adult rat brain VSMC (mainly small pial arteries and arterioles). The cell line has phenotypical and immunohistochemical characteristics of VSMC. These cells were then infected with Schmidt-Ruppin Rous sarcoma virus-strain D, an arian retrovirus to induce immortality. This cell line has proven to be a useful model for studying the specialized biochemical and functional properties of these cells. Primary human cerebral VSMC cells were used at passages 4 and maintained at 37 °C in humidified air containing 5 % CO_2_.

### ROS assay

Cells were plated onto 96-well black clear-bottomed plates (Corning Inc. Life Sciences, Tewksbury, MA) at a density of 50 × 10^3^ cells/well the day prior to experiments. The day of experiments (cells density at near confluency), cells were washed gently for 10-15 seconds with warmed (37 °C) Leibovitz’s media (L-15; no phenol red indicator), loaded with the ROS-sensitive dye Mitotracker Red CM-H_2_XRos (MTR, final concentration of 5 μM; Molecular Probes, Eugene, OR) or dihydroethidium (DHE, final concentration of 10 μM; Molecular Probes) and incubated for 20-30 min. After incubation, cells were washed with warm L-15 media and freshly prepared Aβ peptides (dissolved in L-15 media) were added immediately before measurements at room temperature. In some experiments, cells were co-treated with apocynin (10 μM) and Aβ_1-40_ (2 μM). Fluorescence was measured at room temperature with a plate reader (Synergy HTTR with KC4 software) over 30 min (λ_Ex_ = 475 and λ_Em_ = 645 for MTR, and λ_Ex_ = 520 and λ_Em_ = 610 for DHE).

In some experiments, cells were pre-incubated with heparin (15 U/mL), sodium chlorate (5-50 mM), active or heat denatured (boiled at 100 °C for 20 min) heparinase I (5 Sigma units/mL) and heparinase III (2 Sigma units/mL), chondroitinase AC (10^-1^ IU/mL), or chondroitinase B (10^-1^ IU/mL) in growth media for 2 h prior to analysis. For neutralizing antibody experiments, cells were pre-incubated with antibody (10 μg/mL) in growth media for 2-3 h or co-treated with HJ5-1 antibody (0.3 mg/mL) and Aβ_1-40_ (2 μM).

### Intracellular Ca^2+^ activity assay

Human or rat VSMC were plated onto 96-well black-bottomed plates (Corning Inc. Life Sciences, Tewksbury, MA) at a density of 50 × 10^3^ cells/well the day prior to the experiments. The day of experiments (cell density at near confluency), cells were washed with warmed (37 °C) L-15 media (no phenol red indicator), loaded with the Fura II Ca^2+^-sensitive dye (Invitrogen, Molecular Probes, Eugene, OR; 10 μM), and incubated at 37 °Cfor 20-30 min. After incubation, cells were washed with L-15 media and freshly prepared Aβ peptides (dissolved in L-15 media) were added immediately before measurements. In select experiments, cells were pre-treated with active or heat denatured (boiled at 100 °C for 20 min) heparinase I (5 Sigma units/mL). In other experiments, either an L-type Ca^2+^ channel antagonist (diltiazem or verapamil; 10 μM) or the Ca^2+^ chelator ethylene glycol tetraacetic acid (EGTA; 5 mM) was added to the extracellular media. The addition of ionomycin to each sample well (Sigma-Aldrich, St. Louis, MO; 2 μM) was used as a positive control for intracellular Ca^2+^ signal. Rat VSMC were also treated with aminoethoxydiphenyl borate (2APB, 10 μM), an IP3-receptor inhibitor or ryanodine (1H-Pyrrole-2-carboxylic acid, 10 μM), a Ryanodine receptor inhibitor.

The quantification of intracellular Ca^2+^ influx was performed using ratiometric measurements as previously described by Grynkiewicz et al. [38] with additional corrections for viscosity according to Poenie et al. [39] The pH sensitivity of Fura II Ca^2+^-sensitive dye was calculated using the method of Batlle et al. [40]. A detailed description of the calculations for these measurements can be found in our previous work [41].

### Analysis of Aβ peptides

Aβ_1-40_- and Aβ_1-42_-conditioned media (5 μM) was removed from human cerebral VSMC cultures after a 30 min incubation at 37 °C and fractionated over a size exclusion column (Superdex 200 10/300; 1 mL/min; GE Healthcare Life Sciences, Piscataway, NJ) using fast performance liquid chromatography (FPLC). The eluted fractions were tested for the presence of Aβ using an established enzyme-linked immunosorbent assay (ELISA) [42]. Fractions containing Aβ peptide were further analyzed by sodium dodecyl sulfate (SDS) and native polyacrylamide gel electrophoresis (PAGE) and Western blotting.

### SDS- and native-PAGE

For denaturing conditions, samples were boiled in Laemmli sample buffer (0.125 M Tris [pH 6.8], 4 % SDS, 20 % glycerol, and 10 % β-mercaptoethanol), loaded onto a 16.5 % Tris-Tricine Criterion PreCast gel (BioRad Life Science, Hercules, CA), resolved electrophoretically, and transferred onto a nitrocellulose membrane. Membranes were boiled in phosphate buffered saline for 5 min, blocked with a 5 % (w/v) solution of non-fat dry milk in tris buffered saline with tween (TBST), and then incubated for 16 h at 4 °C in a primary antibody solution (82E1; 1 μg/mL). Following a secondary incubation with a HRP-conjugated goat anti-mouse antibody (Jackson ImmunoResearch, West Grove, PA), the membranes were processed using enhanced chemiluminescence (Clarity Western ECL Substrate; Biorad Life Science, Hercules, CA) and protein bands were detected using the G:BOX iChemi XT imaging system (Syngene, Frederick, MD). For native conditions, samples were mixed with NativePAGE sample buffer (Life Technologies, Carlsbad, CA), loaded onto a Novex 4-20 % Tris-Glycine Native gel (Life Technologies, Carlsbad, CA), resolved electrophoretically, and transferred onto nitrocellulose membranes. The remainder of the procedure was analogous to that performed for denaturing conditions.

### Cell surface area assay

Cells were grown in 48-well culture dishes, washed with warmed L-15 media, and images were acquired with an inverted light microscope (Nikon Eclipse E800; Nikon, Melville, NY). Following a 30-min incubation at 37 °C with varying Aβ preparations, additional light micrographs were obtained. In all instances, the experimenter who acquired the micrographs was blinded to the treatment conditions. All cells were then treated with warm L-15 media containing 100 mM potassium chloride (KCl) and cell surface area was measured at 1, 3, and 5 min after KCL addition, as described earlier [21]. Images were acquired from the same quadrant of each well (near the center of the dish) to minimize variations of cell layering at the culture dish periphery. All micrographs were processed using the Image J software package (National Institute of Health website; www.imagej.nih.gov) and cell surface area was quantified using the automatic threshold function. Data was analyzed as % change in relative surface area units as compared to the vehicle-treated control cells.

### Immunoprecipitation of HSPG

Human VSMC cells were grown to near confluency in 100 mm petri dishes and treated with Aβ_1-40_ (2 μM) or Aβ_40-1_ (2 μM) for 30 min. Cells were washed four times with HBSS and cells were lysed with RIPA buffer. Cell lysates were immunoprecipitated with anti-Heparan Sulphate Proteoglycan (Large) antibody (A7L6, Abcam). Samples were immunoprecipitated using a Protein G immunoprecipitation kit (Roche Applied Sciences, Indianapolis, IN) followed by SDS-PAGE separation and transfer onto nitrocellulose membranes (Millipore Corp). Immunoblotting was performed by incubating the membrane with mouse anti-Aβ antibody (6E10, Sigma). Following incubation the corresponding secondary goat-anti-Rabbit HRP-conjugated antibody (Santa Cruz Biotech) was used to detect immunoreactive product with chemiluminescent kit (BioRad).

### Quantitative Polymerase Chain Reaction (qPCR)

Cells were grown to near confluency in 6-well plates, washed with PBS, and total RNA was isolated using Trizol reagent (Life Technologies, Carlsbad, CA) followed by synthesis of cDNA by reverse transcriptase using the High Capacity cDNA Reverse Transcriptase Kit (Applied Biosystems, Foster City, CA). Sense and antisense oligonucleotides were designed for each HSPG subtype (Additional file 1: Table S1) using a real-time PCR primer design tool (Integrated DNA Technologies, Coralville, IA). qPCR was performed using a 7500 real-time PCR System (Applied Biosystems, Foster City, CA) and the reactions were performed using SYBR Green PCR Master Mix reagents (Applied Biosystems, Foster City, CA). GAPDH expression was used as an internal loading control. Data were analyzed using the delta-delta calculation method to calculate fold change relative to controls [43].

### Statistics

Determination of significance was accomplished by use of a student’s two-tailed t test or ANOVA, depending on the design of the experiment. The level of statistical significance was set at 0.05.

## Results

### Aβ_1-40_ and Aβ_1-42_ induce ROS production via NADPH oxidase

**Fig. 1:**
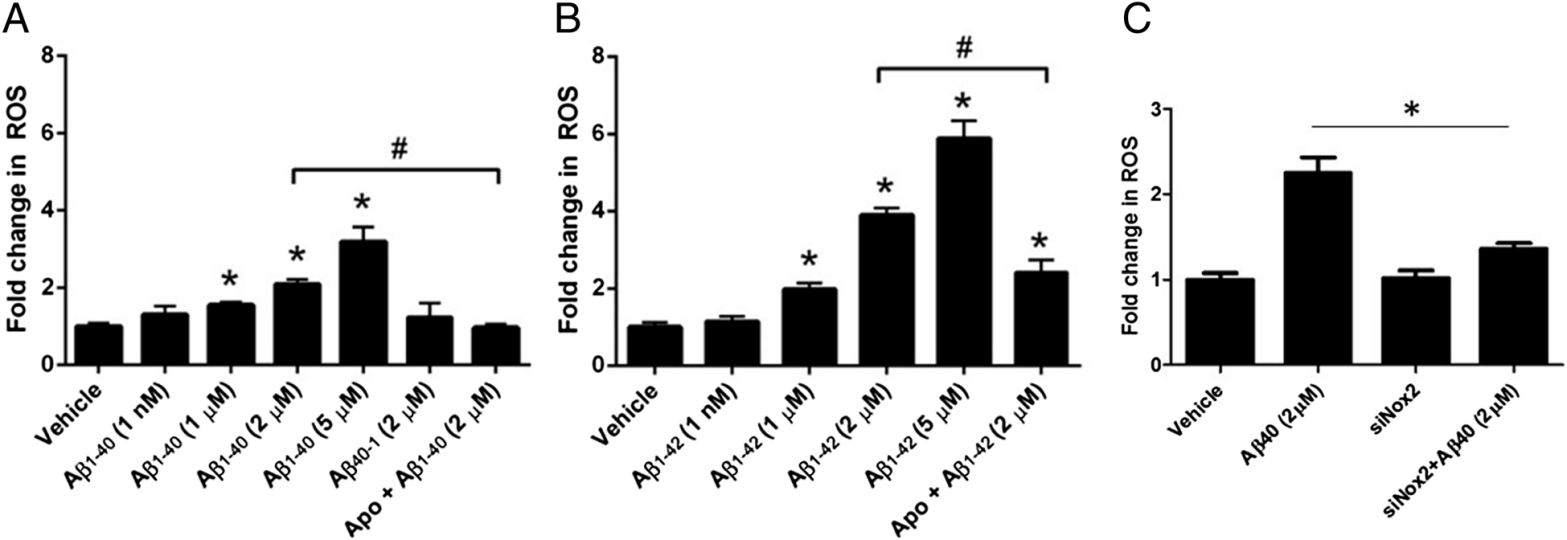
Soluble, monomeric Aβ induces a dose-dependent increase in ROS in primary human cerebral VSMC. VSMC were loaded with Mitotracker Red CM-H_2_XRos (5 μM) and treated with varying concentrations of Aβ_1-40_ (panel **a**) or Aβ_1-42_ (panel **b**). In some cases, cells were treated with a scrambled control peptide (Aβ_40-1_) or co-treated with the NADPH oxidase inhibitor apocynin (Apo; 10 μM, panel **a-b**) or siRNA against Nox2 (panel **c**) and Aβ. Fluorescence was measured after 30 minutes. Results are representative of 3 independent experiments performed in triplicate. *p < 0.05 vs. vehicle-treated control. #p < 0.05 vs. comparison group

Previous reports have demonstrated that Aβ-induced ROS is a critical mediator of CV dysfunction in vitro [44], ex vivo [13], and in vivo [4–7, 17–19]. To examine the effects of Aβ_1-40_ and Aβ_1-42_ on ROS production in primary human cerebral VSMC, these cells were loaded with a fluorescent dye sensitive for detecting mitochondrial ROS (Mitotracker Red CM-H_2_XRos), treated with varying Aβ preparations, and assayed for ROS production after 30 min. We observed a dose-dependent increase in ROS with both Aβ_1-40_ and Aβ_1-42_ at micromolar, but not nanomolar, concentrations (Fig. 1a, b). Treatment with a scrambled control peptide (Aβ_40-1_) did not induce ROS production. Co-treatment of cells with Aβ_1-40_ and the NADPH oxidase inhibitor apocynin reduced ROS generation to baseline levels. Interestingly, at each concentration, Aβ_1-42_ was a more potent inducer of ROS than Aβ_1-40_ in human cerebral VSMC (Fig. 1a, b). Apocynin is a non-specific inhibitor of Nox2. To more directly determine whether Nox2 is involved in Aβ_1-40_ induced ROS production, we performed a targeted genetic knockdown experiment in human VSMC using si-Nox2. We found that genetic Nox2 inhibition significantly reduces Aβ_1−40_-induced ROS (Fig. 1c), which indicates a direct role of Nox2 in Aβ_1-40_-induced ROS production.

Several additional control experiments were performed to determine the cellular consequences of Aβ_1-40_-induced ROS production, to examine the impact of apocynin on baseline ROS production, and to assess the impact of physiological oxygen levels on Aβ_1-40_-induced ROS production. To determine whether Aβ_1-40_-induced ROS production produces toxic damage to cellular components, we quantified lipid oxidation levels following Aβ_1-40_ treatment. Human VSMC were exposed to Aβ_1-40_ for 24 h, followed by assessment of lipid oxidation via measurement of thiobarbituric acid reactive substance (Additional file 2: Figure S1). We found that Aβ_1-40_ significantly increases lipid peroxidation - a finding consistent with true Aβ_1-40_-induced oxidative stress. To assess whether apocynin impacts baseline ROS production in VSMC, we treated human VSMC with apocynin alone (without Aβ_1-40_) and found that baseline ROS levels were not impacted (Additional file 3: Figure S2). To determine if differing levels oxygen impact ROS production in Aβ_1-40_ treated VSMC, we performed an experiment using 10 % oxygen (conditions that are considered physiologic [45]) and an experiment using 1 % oxygen (conditions that are hypoxic) and compared these results to our experiments where VSMCs were grown in humidified air containing 5 % CO_2_ (conditions that are not physiologic, but are very commonly used in the field [13]). We found that Aβ_1-40_ induces significant ROS production in cultured VSMCs under both conditions, but that ROS production was greater with 10 % oxygen (Additional file 4: Figure S3A) than 1 % oxygen (Additional file 4: Figure S3B).

Given the tendency of monomeric Aβ to polymerize into oligomers and fibrils over time, we analyzed VSMC conditioned media from the Aβ_1-40_- and Aβ_1-42_-treated (5 μM) samples after 30 min to assess higher order species. Aβ species from VSMC conditioned media were loaded onto a size exclusion chromatography column and fractionated via FPLC. Aβ-containing fractions were detected using ELISA, and those fractions were further analyzed by SDS- and native-PAGE. We found that the majority of Aβ_1-40_ was in monomeric form with minor amounts (<10 %) of higher order, SDS soluble aggregates (Additional file 5: Figure S4A, B). Similarly, the Aβ_1-42_ sample was mostly in monomeric form with minor amounts (< 15-20 %) of higher order, SDS soluble aggregates (Additional file 5: Figure S4C, D). To specifically differentiate monomers vs. oligomers in the media, we utilized an oligomer-specific anti- Aβ antibody (A11). We found that the majority of Aβ remains as monomers in our experimental conditions; however, we do detect a very small amount of oligomers (Additional file 5: Figure S4E).

### Aβ_1-40_-induced ROS production is attenuated by targeted inhibition of HSPG

HSPG are present in both the extracellular matrix (agrin, perlecan, and collagen XVIII) and the cell surface (glypicans 1-6 and syndecans 1-4) [22]. To assess which HSPG subtypes are present in human cerebral and rat cerebral VSMC, we harvested RNA from cell culture lysates and quantified mRNA using qPCR. In human and rat cerebral VSMC, we found that while all HSPG subtype mRNAs were detectable, they were expressed to very different levels (Additional file 6: Figure S5A, B). While greater levels of agrin, perlecan, glypican 1, syndecan 2, syndecan 3 and keratan sulfate mRNA were present in human vs. rat VSMC, greater levels of collagen XVIII, glypican 3, glypican 4, glypican 6, syndecan 1, syndecan 4 and chondroitin sulphate mRNA were present in rat vs. human VSMC. Given the known interaction of several HSPG subtypes with Aβ [22, 24], we hypothesized that HSPG could be involved in Aβ-induced ROS production.

**Fig. 2:**
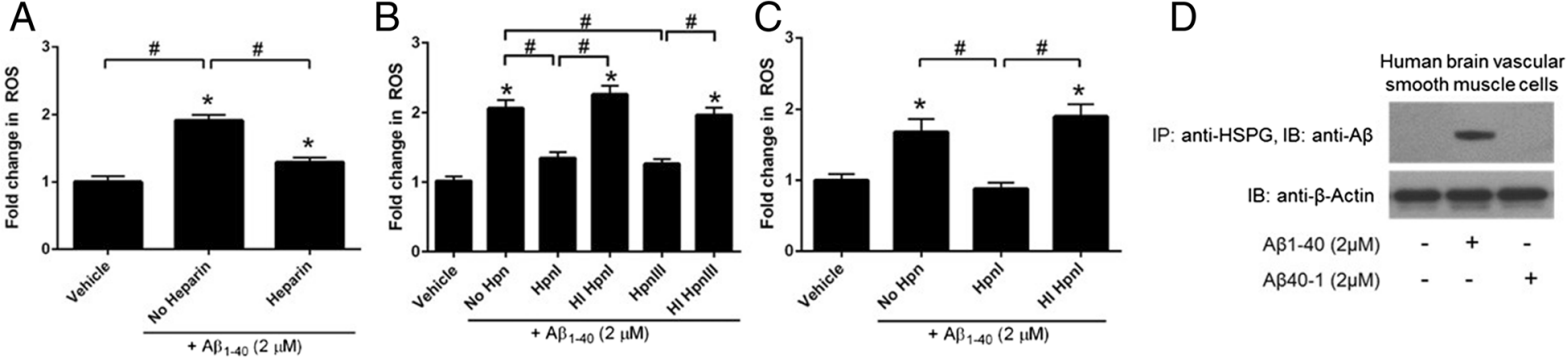
Pharmacological knockdown of HSPG mitigates Aβ_1-40_-induced mitochondrial and cytosolic ROS production in VSMC. Primary human cerebral VSMC were pre-treated with heparin (15 U/mL), heparinase I (HpnI; 5 Sigma U/mL), or heparinase III (HpnIII; 2 Sigma U/mL) for 2 h, washed, loaded with Mitotracker Red CM-H_2_XRos (MTR; 5 μM; panels **a**, **b**) or the cytosolic superoxide-sensitive dye dihydroethidium (10 μM; panel **c**), washed, and treated with Aβ_1-40_. In some cases, cells were pre-treated with heat-inactivated (HI) enzyme (at the same concentration of active enzyme) and washed prior to MTR loading and Aβ treatment. Fluorescence was measured after 30 minutes. To determine if HSPG directly interact with Aβ_1-40,_ human VSMC cells were treated with Aβ_1-40_ for 30 minutes and cell lysates were immunoprecipitated with anti-HSPG antibody and immunoblotted with anti-Aβ antibody (Panel **d**). Results are representative of 3 independent experiments performed in triplicate. *p < 0.05 vs. vehicle-treated control. #p < 0.05 vs. comparison group

Low molecular weight heparin administration has previously been shown to prevent Aβ internalization in cultured cells in vitro [29] and attenuate Aβ-mediated inflammation and neurotoxicity in vivo [46], likely through a HSPG-dependent mechanism [47]. For this reason, we examined whether heparin could mitigate ROS production in cultured human cerebral VSMC. Given that heparin can directly bind Aβ and also exhibits pleotropic cellular effects [48], cells were pre-treated with heparin (15 U/mL), washed, loaded with Mitotracker Red CM-H_2_XRos dye, and treated with Aβ_1-40_ (2 μM). We observed that heparin pre-treatment markedly reduced Aβ-mediated ROS production (Fig. 2a), suggesting a possible contributing role of HSPG.

To more directly implicate HSPG in Aβ-induced ROS production, we pre-treated cells with either active or heat denatured heparinase I (5 Sigma U/mL) and heparinase III (2 Sigma U/mL) followed by washout and ROS measurements. Heparinase is a mammalian endo-β-D-glucuronidase that specifically cleaves heparan sulfate, thereby preventing Aβ-HSPG interactions. We found that pre-incubation with active, but not heat inactivated, heparinase I and III significantly reduced Aβ_1-40_-mediated ROS production (Fig. 2b). We documented similar results after repeating this experiment with VSMC from another source (Cell Biologics, Additional file 7: Figure S6). When these experiments were performed using the fluorescent dye dihydroethidium (DHE; a sensitive indicator of cytosolic superoxide oxygen species), we obtained comparable results (Fig. 2c), suggesting that Aβ-mediated ROS production occurs in both the mitochondria and cytosol.

To determine if HSPG directly interact with Aβ_1-40,_ human VSMC cells were treated with Aβ_1-40_ for 30 minutes and cell lysates were immunoprecipitated with anti-HSPG antibody and immunoblotted with anti-Aβ antibody. Immunoblot showed the presence of Aβ in immunoprecipitated fractions, which suggests direct interaction of Aβ with HSPG (Fig. 2d).

When coupled with the aforementioned pharmacologic experiments targeting HSPG, these data provide further evidence that Aβ_1-40_ plays a key role in mediating VSMC cellular dysfunction.

**Fig. 3:**
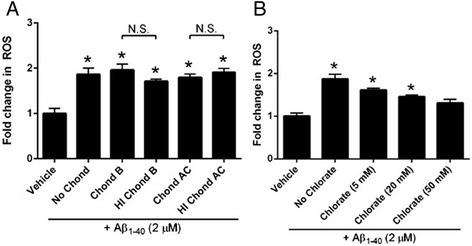
Pharmacological knockdown of other glycosaminoglycan (GAG) family members does not affect Aβ_1-40_-mediated ROS production in VSMC. Primary human cerebral VSMC were pre-treated with chondroitinase B (10^-1^ IU/mL; selectively degrades dermatin sulfate; panel **a**), chondroitinase AC (10^-1^ IU/mL; selectively degrades chondroitin sulfate; panel **a**), or varying concentrations of the sulfation inhibitor sodium chlorate (5-50 mM; panel **b**) for 2 h, washed, loaded with Mitotracker Red CM-H_2_XRos (MTR; 5 μM), and treated with Aβ_1-40_. In some cases, cells were pre-treated with heat-inactivated (HI) enzyme (at the same concentration of active enzyme) and washed prior to MTR loading and Aβ treatment. Fluorescence was measured after 30 minutes. Results are representative of 3 independent experiments performed in triplicate. *p < 0.05 vs. vehicle-treated control. #p < 0.05 vs. comparison group

Finally, we pre-treated cells with increasing concentrations of sodium chlorate, which blocks proper sulfation of HSPG. We found that sodium chlorate reduces Aβ-induced oxidative stress in human VSMC in a dose-dependent fashion (Fig. 3b). This observation is likely caused by the inability of Aβ_1-40_ to effectively interact with non-sulfated HSPG side chains.

In total, our results that Aβ-mediated ROS production is attenuated by three separate HSPG-targeted interventions—heparin that competitively inhibits HSPG; heparinase I or heparanase III that cleave heparan sulfate moieties of HSPG; and sodium chlorate that inhibits sulfation of HSPG side chains—strongly indicate that HSPG are a key mediator of the vascular oxidative stress caused by Aβ.

### Aβ_1-40_-induced ROS production is unaffected by targeted inhibition of other glycosaminoglycan family members

In theory, Aβ could bind either HSPG or other surface proteins, such as chondroitin sulfate proteoglycans and dermatin sulfate proteoglycans. To examine whether enzymatic disruption of other glycosaminoglycan (GAG) family members affects Aβ-mediated ROS elaboration, we pre-treated human cerebral VSMC with active and heat denatured chondroitinase B (which selectively degrades dermatin sulfate) and chondroitinase AC (which selectively degrades chondroitin sulfate) prior to washout and ROS assessment. Our results demonstrate that neither dermatin sulfate nor chondroitin sulfate participate in Aβ-mediated oxygen radical production (Fig. 3a), supporting the notion that Aβ_1-40_ selectively interacts specifically with HSPG to promote oxidative stress.

### Aβ_1-40_-induced VSMC dysfunction is attenuated by targeted inhibition of HSPG

Soluble, monomeric Aβ is a vasoactive peptide that exhibits significant vasoconstrictive properties [6, 49, 50]. The preponderance of data suggests that Aβ_1-40_—the species that primarily comprises the fibrillar deposits of CAA—is a more potent vasoconstrictor than Aβ_1-42_ [51, 52]. To delineate the effects of Aβ treatment on cultured rat cerebral VSMC contractile function, cell surface area was measured after 30 min via light microscopy after treatment with varying Aβ preparations. Of note, rat cerebral VSMC were used instead of human cells because we were unable to reliably quantitate changes in human cerebral VSMC morphology in response to various stimuli given their rigid adherence to the culture dish. However, we verified that rat cerebral VSMC are similar to human cerebral VSMC with respect to Aβ_1-40_-induced ROS production and abrogation of Aβ_1-40_-induced oxidative stress by pharmacological HSPG knockdown (Additional file 8: Figure S7A, B), though the magnitude of ROS production was different (likely the result of a species-specific effect).

**Fig. 4:**
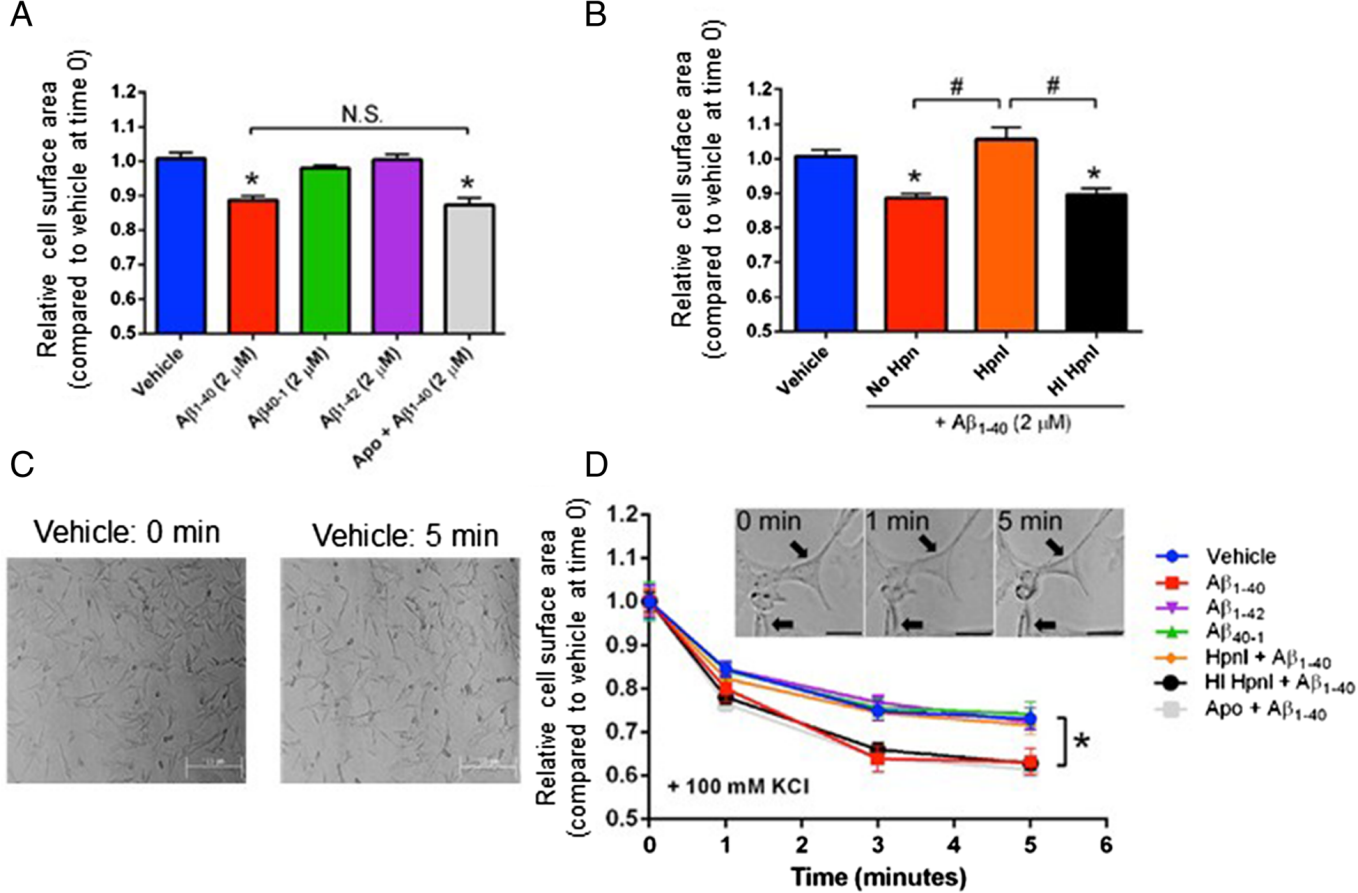
Pharmacological knockdown of HSPG prevents Aβ_1-40_-mediated VSMC constriction. Transformed rat cerebral VSMC surface area was measured via light microscopy 30 minutes after the addition of varying Aβ preparations (panel **a**). In some cases, cells were pre-treated with heparinase I (HpnI; 5 Sigma U/mL) or heat-inactivated heparinase I (HI HpnI; 5 Sigma U/mL) for 2 h, washed, and treated with Aβ_1-40_ (panel **b**). In other cases, cells were co-treated with the NADPH oxidase inhibitor apocynin (10 μM) and Aβ_1−40_. After 30 minutes, cells were treated with KCl (100 mM) and surface area was measured at 1, 3, and 5 minutes (panel **c, d**). Panel **c**: light micrographs at 0, and 5 minutes after vehicle; Inset: light micrographs at 0, 1, and 5 minutes after KCl addition (scale bar = 10 μM). *p < 0.05 vs. vehicle-treated control. #p < 0.05 vs. comparison group. The arrows represent individual cells undergoing a phenotypical change (e.g., vasoconstriction) following Aβ_1-40_ treatment

In agreement with prior studies [21], we observed a 12 % decrease in cell surface area following treatment with Aβ_1-40_, but not with the Aβ_1-42_ or Aβ_40-1_ scrambled peptide (Fig. 4a). Also, co-treatment of cells with Aβ_1-40_ and apocynin did not significantly change VSMC surface area relative to cells treated with Aβ_1-40_ alone. This observation suggests that in rat cerebral VSMC, inhibition of ROS formation does not necessarily prevent Aβ-mediated VSMC constriction. Pre-incubation of cells with active—but not heat denatured—heparinase I enzyme (5 Sigma U/mL) followed by washout and treatment with Aβ_1-40_ effectively reversed the Aβ_1-40_-induced hypercontractile phenotype (Fig. 4b). After 30 minutes, cells were treated with KCl (100 mM) to open cell surface voltage-gated calcium channels [53] and VSMC surface area was measured over an additional 5 minutes. We found that VSMC initially treated with Aβ_1-40_, apocynin + Aβ_1-40_, and heat inactivated heparinase I + Aβ_1-40_ all reduced VSMC surface area after KCl treatment relative to the vehicle-treated control at 3 and 5 minutes (Fig. 4c-d). Conversely, rat cerebral VSMC initially treated with Aβ_1-40_ + heparinase I followed by treatment with KCl responded similarly to the vehicle-treated control at 3 and 5 minutes (Fig. 4c-d).

### Potential role of Ca^2+^ in Aβ_1-40_-induced VSMC dysfunction

**Fig. 5:**
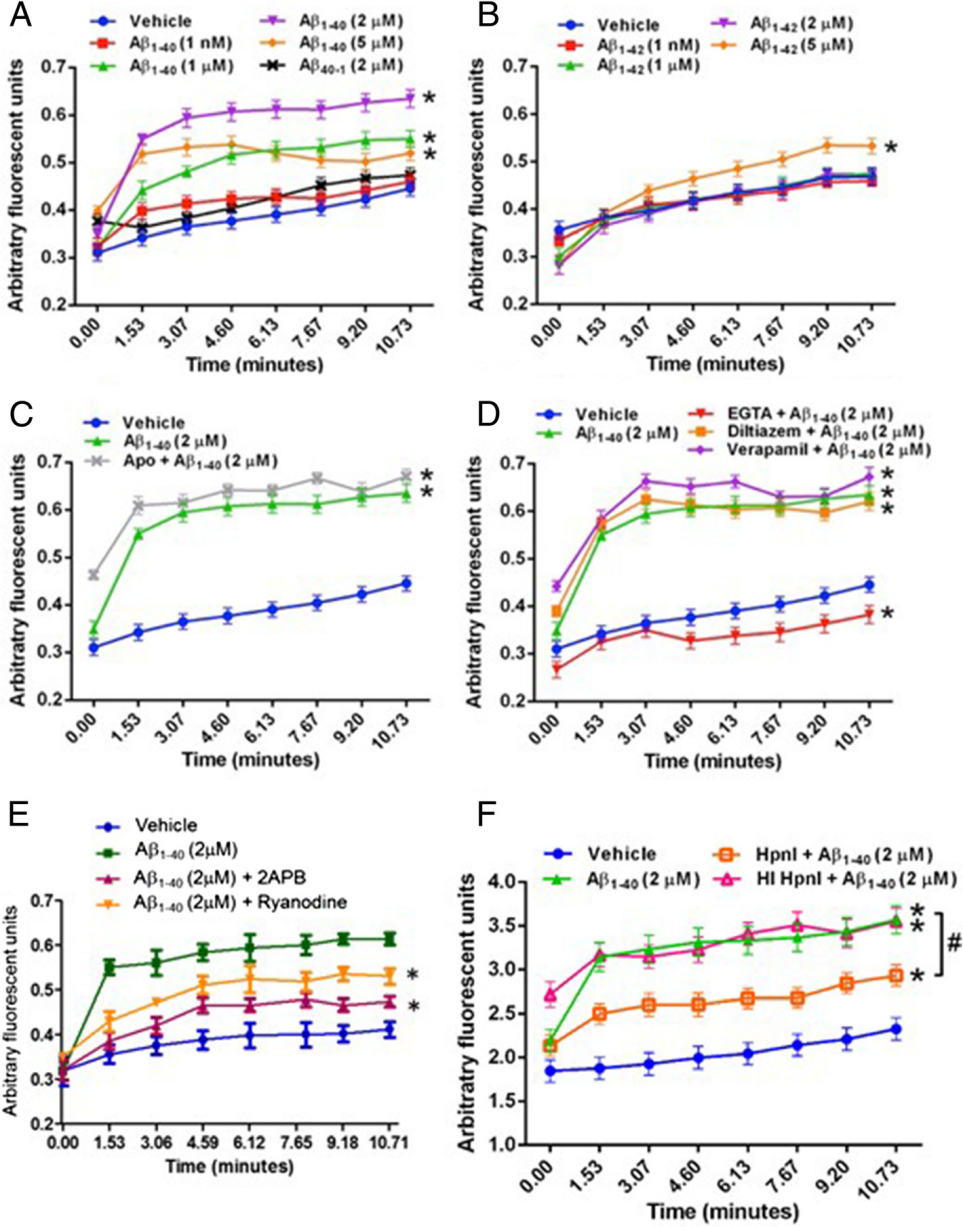
Aβ_1-40_, but not Aβ_1-42,_ induces Ca^2+^ influx into VSMC and this Ca^2+^ influx does not occur through L-type Ca^2+^ channels. Transformed rat cerebral VSMC were loaded with fura II (10 μM) and treated with varying concentrations of Aβ_1-40_ (panel **a**) or Aβ_1-42_ (panel **b**). In some cases, cells were treated with a scrambled control peptide (Aβ_40-1_; panel **a**) or co-treated with the NADPH oxidase inhibitor apocynin (Apo; 10 μM) and Aβ_1-40_ (panel **c**). In other experiments, cells were co-treated with an L-type Ca^2+^ channel antagonist (either verapamil or diltiazem; 10 μM) and Aβ_1-40_, or treated with Aβ_1-40_ in the presence or absence of the Ca^2+^ chelator EGTA (5 mM; panel **d**). Rat VSMC were also treated with aminoethoxydiphenyl borate (2APB, 10 μM), an IP3-receptor inhibitor or ryanodine (1H-Pyrrole-2-carboxylic acid, 10 μM), a Ryanodine receptor inhibitor (panel **e**). In some experiments, cells were pre-treated with active or heat-inactivated heparinase I (HpnI; 5 Sigma U/mL), washed, loaded with fura II, and treated with Aβ_1−40_ (panel **f**). Fluorescence was measured over ~10 minutes. Results are representative of 3 independent experiments performed in triplicate. *p < 0.05 vs. vehicle-treated control

To examine the role of Ca^2+^ in the regulation of Aβ_1-40_- and Aβ_1-42_-induced ROS production and Aβ_1-40_-induced VSMC hypercontractility, we utilized the Ca^2+^-sensitive dye Fura II to measure changes in intracellular Ca^2+^ levels. Rat cerebral VSMC were loaded with Fura II, washed, and then treated with varying concentrations of Aβ_1-40_ and Aβ_1-42_. We observed that intracellular Ca^2+^ influx increased as a function of Aβ_1-40_ concentration over ~10 minutes, with the maximum level of intracellular Ca^2+^ achieved after treatment with Aβ_1-40_ at 2 μM concentrations (Fig. 5a). Interestingly, even at higher concentrations, the level of intracellular Ca^2+^ achieved after treatment of cells with Aβ_1-42_ was well below that of cells treated with Aβ_1-40_ (Fig. 5b). Co-treatment of rat cerebral VSMC with the NADPH oxidase inhibitor apocynin did not reverse Aβ_1-40_-mediated Ca^2+^ influx (Fig. 5c).

Next, we examined the route(s) by which Ca^2+^ enters the intracellular space following Aβ_1-40_ treatment in VSMC. We found that Ca^2+^ does not enter through L-type Ca^2+^ channels given that the L-type Ca^2+^ channel blockers verapamil and diltiazem did not decrease intracellular Ca^2+^, but co-incubation with the extracellular Ca^2+^ chelating agent EGTA produced a marked decrement in intracellular Ca^2+^ (Fig. 5d). We did find that 1) treatment with aminoethoxydiphenyl borate (2APB), an IP3-receptor inhibitor, decreased the Aβ_1-40_-induced rise in intracellular Ca2+ (Fig. 5e) and 2) treatment with ryanodine (1H-Pyrrole-2-carboxylic acid), a Ryanodine receptor inhibitor, induced a small inhibition of the Aβ_1-40_-induced rise in intracellular Ca2+ (Fig. 5e). In total, these data indicate Aβ_1-40_ treatment likely stimulates the release of Ca2+ into the intracellular space via IP-3 receptors. Non-specific Ca2+ entry through the cell membrane is also possible.

We also examined whether HSPG mediate the Ca2+ influx observed following Aβ_1-40_ treatment in rat and human VSMC. We found that pre-incubation of rat cerebral VSMC with active, but not heat-inactivated, heparinase I attenuated Aβ_1-40_-induced Ca^2+^ influx (Fig. 5f). Using ratiometric quantification of intracellular Ca^2+^, we observed that treatment of rat cerebral VSMC with Aβ_1-40_ (2 μM) resulted in a 216.1 ± 13.0 nM increase in intracellular Ca^2+^ as compared to a 58.0 ± 3.7 nM increase with vehicle treatment alone (p < 0.001). Pre-treatment of rat cerebral VSMC with active heparinase I resulted in a significant reduction of Aβ_1-40_-induced Ca^2+^ influx as compared to pre-treatment with vehicle alone (91.6 ± 3.3 vs. 216.1 ± 13.0 nM, respectively; p < 0.001), but did not completely return the level of intracellular Ca^2+^ concentration to that of vehicle treatment alone (91.6 ± 3.3 vs. 58.0 ± 3.7 nM, respectively; p < 0.001). Notably, pre-treatment of rat cerebral VSMC with heat-inactivated heparinase I resulted in an increase in Aβ_1-40_-induced Ca^2+^ influx as compared to pre-treatment with active heparinase I (180.4 ± 8.4 vs. 91.6 ± 3.3 nM, respectively; *p* < 0.001). We also found a similar (albeit less pronounced) HSPG-mediated effect of Aβ_1-40_ on Ca^2+^ influx in human VSMC (Additional file 9: Figure S8). Collectively, these data suggest that pharmacological interference with HSPG attenuates Aβ_1-40_-induced increases in intracellular Ca^2+^ in rat and human cerebral VSMC.

**Fig. 6:**
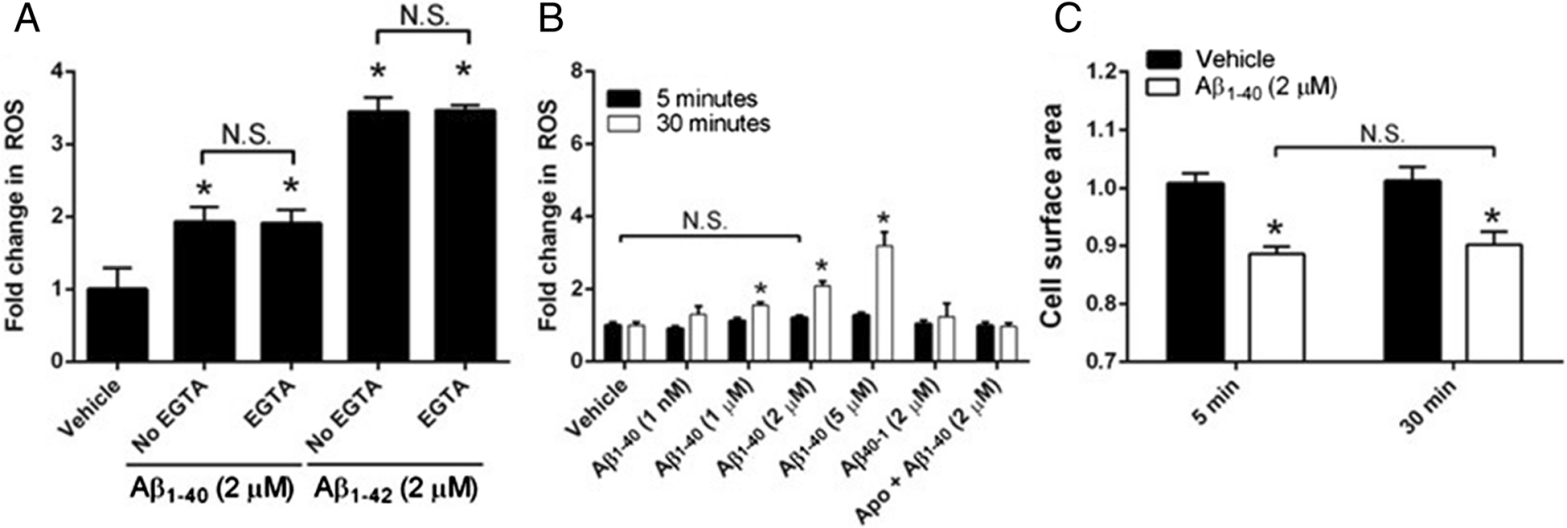
Aβ_1-40_- and Aβ_1-42_-mediated ROS production is not dependent on intracellular Ca^2+^ influx. Transformed rat cerebral VSMC were loaded with Mitotracker Red CM-H_2_XRos (5 μM) and treated with either Aβ_1-40_ or Aβ_1-42_ in the presence or absence of EGTA (5 mM) in the extracellular media (panel **a**). Aβ_1-40_-mediated ROS production in VSMC was measured after 5 minutes and compared with ROS production after 30 minutes (panel **b**). Aβ_1-40_-mediated changes in VSMC surface area were measured after 5 minutes and compared with VSMC surface area after 30 minutes (panel **c**)

To determine whether intracellular Ca^2+^ influx is required for Aβ_1-40_ and Aβ_1-42_-induced ROS production, we co-incubated rat cerebral VSMC with EGTA and assayed for ROS production after 30 minutes following treatment with either Aβ_1-40_ or Aβ_1-42_. Our results show that Ca2+ influx may not be essential for either Aβ_1-40_- or Aβ_1-42_-induced ROS production as EGTA failed to inhibit amyloid-induced ROS (Fig. 6a). We found that Aβ_1-40_-mediated ROS production requires incubation of at least ~30 minutes (Fig. 6b), however, Aβ_1-40_-induced Ca^2+^ influx and VSMC hypercontractility occur within 5 minutes following Aβ_1-40_ treatment (Fig. 6c).

## Discussion and conclusions

**Fig. 7:**
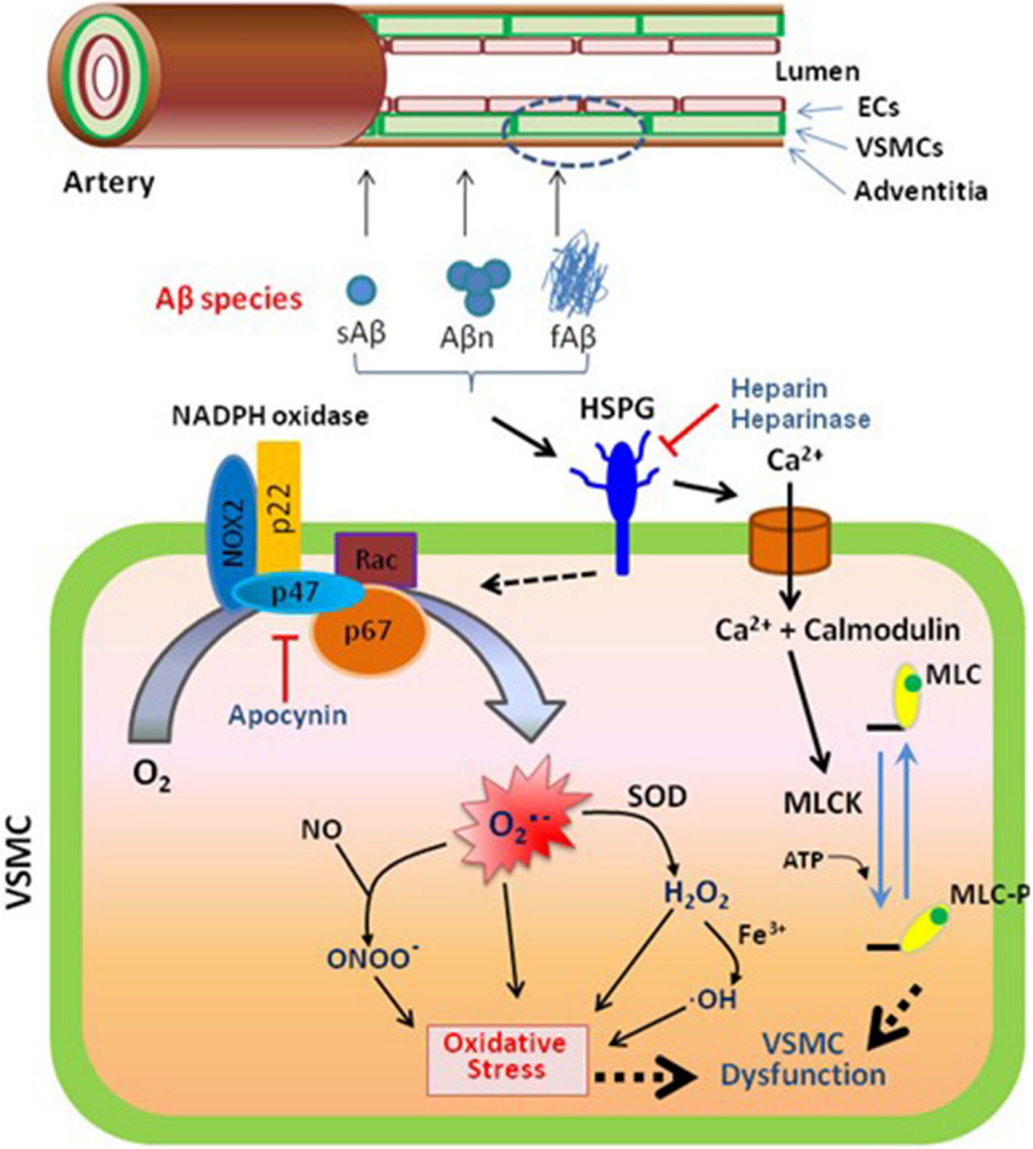
Schematic illustrating the cascade of intracellular events culminating in Aβ-induced Ca^2+^ influx, ROS production, and VSMC contractility. Aβ (in either monomeric, oligomeric, or fibrillar form) may interact with cell surface or extracellular matrix HSPG, leading to intracellular Ca^2+^ influx (early event, ~2 mins) and ROS production (later event, ~30 mins). Toxic ROS species may directly damage the VSMC contractile machinery leading to a hypercontractile phenotype. Also, via an independent or interdependent pathway, intracellular Ca^2+^ may bind to calmodulin to activate myosin light chain kinase (MLCK) and facilitate VSMC contraction. Interference with Aβ-HSPG binding via treatment with heparin or heparinase can mitigate these toxic effects of Aβ

Our results demonstrate that (1) Aβ_1-40_ and Aβ_1-42_ (but not the scrambled peptide Aβ_40-1_) induce ROS production in cultured human cerebral VSMC in a dose-dependent fashion; (2) Aβ_1-40_- and Aβ_1-42_-induced ROS production is mediated downstream by NADPH oxidase activation, as co-treatment with the NADPH oxidase inhibitor apocynin or co-treatment with siRNA targeting Nox2 mitigated Aβ-induced ROS production in cultured human cerebral VSMC; (3) Aβ_1-40_- and Aβ_1-42_-induced ROS production is mediated via HSPG, as pre-treatment with heparin, heparinase (I and III), and sodium chlorate prevents Aβ-induced ROS production in cultured human cerebral VSMC and knockdown of other GAG family members (as well as antibody-mediated interference with other known Aβ binding partners on the cell surface) did not recapitulate this protective effect; (4) Aβ_1-40_, but not Aβ_1-42_, induces a hypercontractile phenotype in cultured rat VSMC—an effect that is dependent on HSPG but not NADPH oxidase, as Aβ_1-40_-induced VSMC hypercontractility was reversed when cells were pre-incubated with heparinase I but not when cells were co-treated with apocynin; and (5) Aβ_1-40,_ but not Aβ_1-42_, induces HSPG-dependent Ca^2+^ influx that appears to underlie the pathologic effect of Aβ_1-40_ on VSMC function, as Aβ_1-40_ causes Ca^2+^ influx into cultured rat VSMC that coincides temporally with Aβ_1-40_-induced VSMC hypercontractility. Importantly, both Aβ_1-40_-induced VSMC dysfunction and Aβ_1-40_-induced Ca^2+^ influx appear mediated via HSPG, as pre-incubation with heparinase I attenuated both events. Overall, our data not only confirm past studies that show Aβ species induce vascular oxidative stress via NADPH oxidase, but extend upon them by shedding important mechanistic insight into the upstream molecular events by which Aβ-induced ROS production and Aβ-induced VSMC dysfunction occur. Specifically, our data strongly implicate HSPG as a key mediator of Aβ_1-40_- and Aβ_1-42_-induced VSMC oxidative stress, Aβ_1-40_-induced VSMC hypercontractility, and Aβ_1-40_-induced Ca^2+^ influx (Fig. 7).

These results are important for a number of reasons. First, multiple lines of evidence indicate that vascular pathologies including Aβ have significant and independent contributions to the dementia of AD, a realization that has led the American Heart Association [54], the Alzheimer’s Association [55], and the National Institute on Neurological Disorders and Stroke [56] to prioritize studies investigating the nature and mechanisms of vascular contributors to dementia. Second, while oxidative stress has been linked to the CV dysfunction caused by Aβ species in a variety of in vitro [13], ex vivo [13], and in vivo [4–7, 17–19] experimental paradigms, the upstream molecular events leading to this vascular oxidative stress are poorly understood. Identification of these events would likely lead to discovery of novel molecules that that could serve as new therapeutic targets for patients with AD, CAA, or both. Our results implicating HSPG as a key mediator of Aβ-induced oxidative stress and Aβ_1-40_-induced VSMC dysfunction strongly suggest that this cell surface molecular complex represents a new pharmacologic target deserving of additional investigation. Third, while our study concentrated on the pathologic effects of two monomeric forms of Aβ (Aβ_1-40_ and Aβ_1-42_) on one vascular cell type (VSMC), our results may well have mechanistic implications on other forms of Aβ-induced CV dysfunction including that caused by vascular endothelial cell (VEC) dysfunction and that caused by higher order Aβ species. Support for the former comes from in vitro, ex vivo, and in vivo studies that implicate ROS in Aβ-induced VEC dysfunction [13, 17, 57] and VEC-mediated vasomotor impairment; [13, 17, 57–59] support for the latter comes from in vitro and in vivo studies demonstrating that oligomeric Aβ (at least in neurons) [60] and fibrillar Aβ (in cerebral vessels and neurons) [18, 61–65] cause even greater degrees of oxidative stress. Therefore, while elevated levels of soluble Aβ—and their attendant vascular consequences including altered CV reactivity [6] and impaired CBF [49, 50]—are present in the early stages of AD when fibrillar Aβ in the form of neuritic plaques and CAA have yet to develop to a significant degree, the mechanisms elucidated in our study may very well exert their greatest impact on the later stages of AD when higher order Aβ species are much more abundant. Future studies will be required to examine this intriguing possibility.

One interesting and at first counterintuitive observation from our results is that while exogenous Aβ_1-42_ application induces ROS production somewhat more effectively than Aβ_1-40_ in cultured human and rat VSMC, only Aβ_1-40_ generates a hypercontractile phenotype in rat VSMC. The most plausible explanation for this finding is that Aβ_1-40_ induces an influx of intracellular Ca^2+^ much more effectively than Aβ_1-42_ (a notion supported by our data; see Figs. 5 and 6), and that Aβ-mediated ROS production and Aβ-mediated Ca^2+^ influx may contribute to CV dysfunction via independent, or interdependent, processes. Our data support a scenario whereby Aβ monomers interact with cell surface and/or extracellular matrix HSPG leading to an early intracellular Ca^2+^ influx (~2 min) and a later elaboration of ROS species (~30 min) (Fig. 6). These toxic ROS may subsequently direct damage to the VSMC contractile machinery, thereby leading to a hypercontractile phenotype. In addition, through an independent or interdependent process, intracellular Ca^2+^ may contribute to Aβ-induced VSMC dysfunction by binding to calmodulin and activate myosin light chain kinase to facilitate VSMC contraction (Fig. 7). Determining which of these two processes is the primary driver of Aβ-induced VSMC dysfunction, and assessing whether either process is dependent on the other will require additional experiments. However, our initial studies indicate the following: 1) Ca2+ influx does not appear necessary for either Aβ_1-40_- or Aβ_1-42_-induced ROS production in VSMC (Fig. 6a); and 2) Aβ_1-40_-induced VSMC hypercontractility appears to temporally coincide with Aβ_1-40_-induced Ca^2+^ influx and precede significant Aβ_1-40_-mediated ROS production (Fig. 6b, c). As such, it may be that intracellular Ca^2+^ activity—rather than intracellular ROS—is the primary driver Aβ_1-40_-induced VSMC dysfunction.

That interference with Aβ-HSPG binding via treatment with heparin, heparinase, or sodium chlorate mitigated both of these Aβ-induced toxic effects argues strongly for the concept that HSPG-directed therapies carry promise as a new approach towards combating the consequences of Aβ-induced CV dysfunction in patients with AD, CAA, or both. Another interesting observation from our data was that apocynin co-treatment of VSMC reduced Aβ-mediated ROS production (Fig. 1) but did not rescue these cells from a hypercontractile phenotype (Fig. 4). These data seem to conflict with our prior observation in explanted rat cerebral arterioles that strategies aimed to reduce ROS were effective in reversing the Aβ-mediated hypercontractile response [13]. Most likely, this observation relates to the differing experimental models utilized in our two studies. In the present report, we utilized isolated VSMC monocultures that permit direct measurement of VSMC function, while in our former study [13] we employed isolated cerebral arterioles that permit assessment of both VEC-dependent as well as VEC-independent vasomotor function. It is plausible that Aβ-mediated ROS production has a greater functional impact on VEC than on VSMC, which explain our [13] and others [17, 19] past findings that vascular oxidative stress is an important mediator of Aβ-induced CV dysfunction in the setting of intact vessels (ex vivo and in vivo).

Our study has several limitations. First, our experiments were performed in tissue culture and may not be generalized to the ex vivo or in vivo settings. Second, our experiments focused solely on the role of Aβ-mediated toxic effects in cultured VSMC. To address both of these limitations, we are currently performing experiments to determine the role of HSPG in the oxidative stress and functional consequences of Aβ in cultured VEC and Aβ applied to isolated cerebral vessels. Third, while fibrillar Aβ in the form of CAA is commonly present in the smooth muscle cell layer of cerebral arterioles, it is possible that monomeric Aβ may not be present in this compartment at μM concentrations. While the concentration of Aβ in the serum and cerebrospinal fluid has been estimated in the nM range [66], recent evidence suggests soluble Aβ species are in a dynamic equilibrium with fibrillar Aβ deposits in brain and cerebral vessels [35] which would allow for higher local concentrations of Aβ in brain regions immediately surrounding fibrillar Aβ deposits. Therefore, it is entirely possible that the local concentration of monomeric Aβ in the perivascular space is significantly higher (e.g., μM, or even mM), thereby validating the physiological relevance of the Aβ concentrations that we chose for our experiments.

## Additional files

Additional file 1: Table S1. Heparin sulfate proteoglycan (HSPG) primer pairs for quantitative polymerase chain reaction (qPCR). (DOCX 20 kb) Additional file 2: Figure S1. Soluble, monomeric Aβ_1-40_ induces lipid oxidation in human VSMC. Human VSMC were exposed to Aβ_1-40_ for 24 h, followed by assessment of lipid oxidation via measurement of thiobarbituric acid reactive substance (TBARS). In parallel experiments primary human cerebral VSMC were also pre-treated with heparinase I (HpnI; 5 Sigma U/mL). Results are representative of 3 independent experiments performed in triplicate. *p < 0.05 vs. vehicle-treated control. #p < 0.05 vs. comparison group. (JPEG 28 kb) Additional file 3: Figure S2. Apocynin does not impact baseline ROS production in VSMC. Human VSMC were loaded with Mitotracker Red CM-H_2_XRos (5 μM) and treated with Aβ_1-40_. In some cases, cells were co-treated with the NADPH oxidase inhibitor apocynin (Apo; 10 μM). Fluorescence was measured after 30 minutes. Results are representative of 3 independent experiments performed in triplicate. *p < 0.05 vs. vehicle-treated control. #p < 0.05 vs. comparison group. (JPEG 31 kb) Additional file 4: Figure S3. HSPG mitigates Aβ_1-40_-induced mitochondrial and cytosolic ROS production in VSMC under physiological oxygen concentration. To determine if differing levels oxygen impact ROS production in Aβ_1-40_ treated VSMC, cells were kept in 10 % oxygen (Panel A) or 1 % oxygen (conditions that are considered hypoxic; Panel B) in cell culture incubator with % 5 CO_2_. Primary human cerebral VSMC were pre-treated with heparin (15 U/mL), heparinase I (HpnI; 5 Sigma U/mL), or heparinase III (HpnIII; 2 Sigma U/mL) for 2 h, washed, loaded with Mitotracker Red CM-H_2_XRos, washed, and treated with Aβ_1-40_. In some cases, cells were pre-treated with heat-inactivated (HI) enzyme. Fluorescence was measured after 30 minutes. Results are representative of 3 independent experiments performed in triplicate. *p < 0.05 vs. vehicle-treated control. #p < 0.05 vs. comparison group. (JPEG 70 kb) Additional file 5: Figure S4. Aβ_1-40_ and Aβ_1-42_ remain mostly in soluble, monomeric form after 30 minute incubation in L-15 media. Conditioned media from Aβ_1-40_- (panels A, B) and Aβ_1-42_-treated VSMC (5 μM) was loaded onto a size exclusion column and fractionated via fast performance liquid chromatography (FPLC). Aβ-containing fractions were identified using enzyme-linked immunosorbent assays (ELISA) and higher-order Aβ species were separated from monomer (4 kDa) by SDS- (panels A, C) and native- (panels B, D) polyacrylamide gel electrophoresis (PAGE) with Western blotting. Aβ_1-40_ and Aβ_1-42_ peptides were detected using the anti-Human Aβ mouse 82E1 monoclonal antibody (1 μg/mL). To specifically differentiate monomers vs. oligomers in the media, we performed dot blot using oligomer-specific anti- Aβ antibody (A11; Panel E). (JPEG 75 kb) Additional file 6: Figure S5. HSPG subtype mRNA is present in primary human cerebral and transformed rat cerebral VSMC. HSPG subtype mRNA was harvested from primary human cerebral as well as transformed rat cerebral VSMC and measured using quantitative polymerase chain reaction (qPCR). Extracellular matrix HSPG include agrin, perlecan, and collagen XVIII; cell surface HSPG include glypicans 1-6 and syndecans 1-4 (Panel A). In addition we also performed qPCR for chondroitin sulphate, dermatan sulphate and keratan sulfate (Panel B). *p < 0.05 vs. comparison group. (JPEG 90 kb) Additional file 7: Figure S6. Human VSMC cells from second source conform that HSPG mitigates Aβ_1-40_-induced mitochondrial and cytosolic ROS production in VSMC. Primary human cerebral VSMC from other source (Cell Biologics, Chicago, IL) were pre-treated with heparin (15 U/mL), heparinase I (HpnI; 5 Sigma U/mL), or heparinase III (HpnIII; 2 Sigma U/mL) for 2 h, washed, loaded with Mitotracker Red CM-H_2_XRos (MTR; 5 μM), washed, and treated with Aβ_1-40_. In some cases, cells were pre-treated with heat-inactivated (HI) enzyme. Fluorescence was measured after 30 minutes. Results are representative of 3 independent experiments performed in triplicate. *p < 0.05 vs. vehicle-treated control. #p < 0.05 vs. comparison group. (JPEG 42 kb) Additional file 8: Figure S7. Transformed rat cerebral VSMC behave similarly to human cerebral VSMC with respect to Aβ_1-40_-induced ROS production and mitigation of Aβ_1-40_-induced oxidative stress by pharmacological knockdown of HSPG with heparinase. Rat cerebral VSMC were loaded with Mitotracker Red CM-H_2_XRos (5 μM) and treated with varying concentrations of Aβ_1-40_ or a scrambled control peptide (Aβ_40-1_; panel A). In some experiments, cells were pre-treated with heparinase (or heat-inactivated enzyme) for 2 h, washed, and then treated with Aβ_1-40_ (2 μM) (panel B). Fluorescence was measured after 30 minutes. Results are representative of 3 independent experiments performed in triplicate. Apo = apocynin. Hpn = heparinase. HI = heat inactivated. *p < 0.05 vs. vehicle-treated control. #p < 0.05 vs. comparison group. (JPEG 56 kb) Additional file 9: Figure S8. Human VSMC cells also show Aβ_1-40_ induces HSPG-mediated Ca^2+^ influx. Primary human VSMC were loaded with fura II (10 μM) and treated with varying concentrations of Aβ_1-40_. In some experiments, cells were pre-treated with active heparinase I (HpnI; 5 Sigma U/mL), washed, loaded with fura II, and treated with Aβ_1−40_. Fluorescence was measured over ~10 minutes. Results are representative of 3 independent experiments performed in triplicate. *p < 0.05 vs. vehicle-treated control. (JPEG 44 kb)

## Abbreviations

AD: Alzheimer’s disease
Aβ: Amyloid Beta
CAA: cerebral amyloid angiopathy
CBF: cerebral blood flow
CSA: cell surface area
CV: cerebrovascular
DHE: Dihydroethidium
EGTA: ethylene glycol tetraacetic acid
ELISA: enzyme-linked immunosorbent assay
FPLC: fast performance liquid chromatography
GAG: glycosaminoglycan
HSPG: heparan sulfate proteoglycans
MTR: mitotracker red CM-H2Xros
NADPH oxidase: nicotinamide adenine dinucleotide phosphate-oxidase
PAGE: polyacrylamide gel electrophoresis
qPCR: quantitative polymerase chain reaction
ROS: reactive oxygen species
VEC: vascular endothelial cells
VSMC: vascular smooth muscle cells
