# Supplementary material for: Heparan sulfate proteoglycans mediate Aβ-induced oxidative stress and hypercontractility in cultured vascular smooth muscle cells
Source: Mol Neurodegener. 2016 Jan 22;11:9. doi: 10.1186/s13024-016-0073-8 (PMC4722750; doi:10.1186/s13024-016-0073-8)
Supplement: Additional file 1: Table S1. — Heparin sulfate proteoglycan (HSPG) primer pairs for quantitative polymerase chain reaction (qPCR). (DOCX 20 kb) [file 13024_2016_73_MOESM1_ESM.docx]

| ***Additional file 1: Table S1:*** *Heparin sulfate proteoglycan (HSPG) primer pairs for quantitative polymerase chain reaction (qPCR)* | | | | | | |
| --- | --- | --- | --- | --- | --- | --- |
|  | | | | | | |
|  | *Human* | | *Rat* |  |  |  |
|  | *Sense* | *Anti-sense* | *Sense* | *Anti-sense* | *Genbank accession #* | *Ref.* |
|  |  |  |  |  |  |  |
| ****GAPDH*** | aca tcg ctc aga cac cat g | tgt agt tga ggt caa tga agg g | cca tca acg acc cct tca tt | gac cag ctt ccc att ctc ag | Human: NM 002046  Rat: NM 017008 |  |
| ***Agrin*** | gat tct cag gac cgc act g | gtt caa agt ggt tgc tct gc | tcc tca gca act aca aac ctg | caa agc cac ata aca ttc ccc | Human: NM 198576  Rat: NM 175754 |  |
| ***Perlecan*** | cat aga gac cgt cac agc aag | agg gct cgg aaa taa acc atc | ccc tgg caa cag ctt cta | atg gcc atc ctg tag tcc aa | Human: NM 005529  Rat: N/A | (1) |
| ***Collagen XVIII*** | caa tgt gtt tgc tga gtc cag | gaa agt caa acg gaa act gcc | gcc cgc atc ttt tct ttc g | cgt ctc aca gta gct ctc cat ca | Human: NM 030582  Rat: N/A | (1) |
| ***Syndecan 1*** | gaa gat caa gat ggc tct ggg | gtt ctg gag acg tgg gaa tag | ctg aag acc aag atg gct ctg | gtt ctg gag ctg tgg gtg | Human: NM 001006946  Rat: NM 013026 |  |
| ***Syndecan 2*** | cca gcc gaa gag gat aca aat g | tct cat gcg ata cac caa cag | atc tgt tca agc gga cgg | cag gat gag gaa aat ggc aaa g | Human: NM 002998  Rat: NM 013082 |  |
| ***Syndecan 3*** | aga gta tcc tgg agc gga ag | cga tag atg agc agt gtg aac | cgg tag atg agc aac gtg ac | gaa gag cat act aga gcg gaa g | Human: NM 014654  Rat: NM 053893 |  |
| ***Syndecan 4*** | tga ctt tga gct gtc tgg c | agt ttc ttg ggt tcg gtg g | gga cga tga aga cgc tgg | ggg atg tgg tta tct agt ggc | Human: NM 002999  Rat: NM 012649 |  |
| ***Glypican 1*** | gac tat tgc cga aat gtg ctc | gct gcc gat gac act ctc | cga agt ccg cca gat cta cg | atc ctg cag tgt acg ctc c | Human: NM 002081  Rat: NM 030828 | (2) |
| ***Glypican 2*** | ctc act tat gcc ctg cca g | ccg tca gct caa agg aaa ag | tta ctc ggc tca ctt caa ctg | gct tcg ctg acc aca ttt c | Human: NM 152742  Rat: NM 138511 |  |
| ***Glypican 3*** | tga aga tga gtg cat tgg agg | tgc tta tct cgt tgt cct tcg | tca gtt taa cct cca tga gct g | ggc ttt tat cca caa ctt tac cc | Human: NM 001164617  Rat: NM 012774 |  |
| ***Glypican 4*** | agg aaa cgg caa tga gga tg | gac gaa gga tca gta tgt ctg g | tga gaa tga gga tga ctg ttg g | gcc atg att tga cga agg atc | Human: NM 001448  Rat: NM 001014108 |  |
| ***Glypican 5*** | cag gaa gtg gag aag tca aga g | agt gtc tgt ttg atg gat ttg c | gct cct tgg aag agc tgt cg | aag gcc gca aat ctt att ca | Human: NM 004466  Rat: NM 001107285 | (3) |
| ***Glypican 6*** | att atg gct ctc cgt gtg atg | aca aac tca aac tcc gtg gg | cac ggt tgt gtc aag gca taa g | cag tga aga ggt cct gga ata c | Human: NM 005708  Rat: XM 001077762 |  |
|  | | | | | | |
| *GAPDH = Glyceraldehyde 3-phosphate dehydrogenase  N/A = Not available | | | | | | |

**1.** Shibata M, Shigematsu T, Hatamura I, et al. Reduced Expression of Perlecan in the Aorta of Secondary Hyperparathyroidism Model Rats with Medial Calcification. *Renal Failure.* 2010;32(2):214-223.

**2.** Chernousov MA, Rothblum K, Stahl RC, Evans A, Prentiss L, Carey DJ. Glypican-1 and α4 (V) collagen are required for Schwann cell myelination. *The Journal of Neuroscience.* 2006;26(2):508-517.

**3.** Okamoto K, Tokunaga K, Doi K, et al. Common variation in GPC5 is associated with acquired nephrotic syndrome. *Nature genetics.* 2011;43(5):459-463.
