# Supplementary figures and images for: Heparan sulfate proteoglycans mediate Aβ-induced oxidative stress and hypercontractility in cultured vascular smooth muscle cells
Source: Mol Neurodegener. 2016 Jan 22;11:9. doi: 10.1186/s13024-016-0073-8 (PMC4722750; doi:10.1186/s13024-016-0073-8)

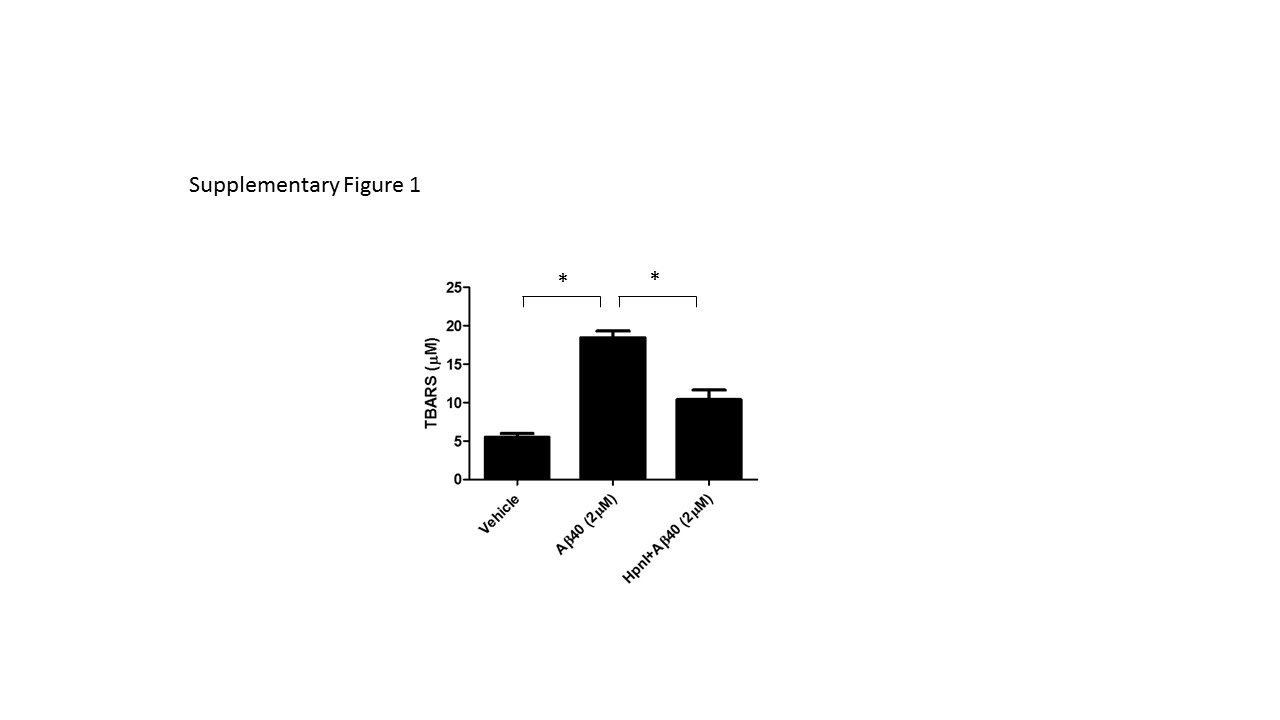

Supplement: Additional file 2: Figure S1. — Soluble, monomeric Aβ1-40 induces lipid oxidation in human VSMC. Human VSMC were exposed to Aβ1-40 for 24 h, followed by assessment of lipid oxidation via measurement of thiobarbituric acid reactive substance (TBARS). In parallel experiments primary human cerebral VSMC were also pre-treated with heparinase I (HpnI; 5 Sigma U/mL). Results are representative of 3 independent experiments performed in triplicate. *p < 0.05 vs. vehicle-treated control. #p < 0.05 vs. comparison group. (JPEG 28 kb) [file 13024_2016_73_MOESM2_ESM.jpeg]

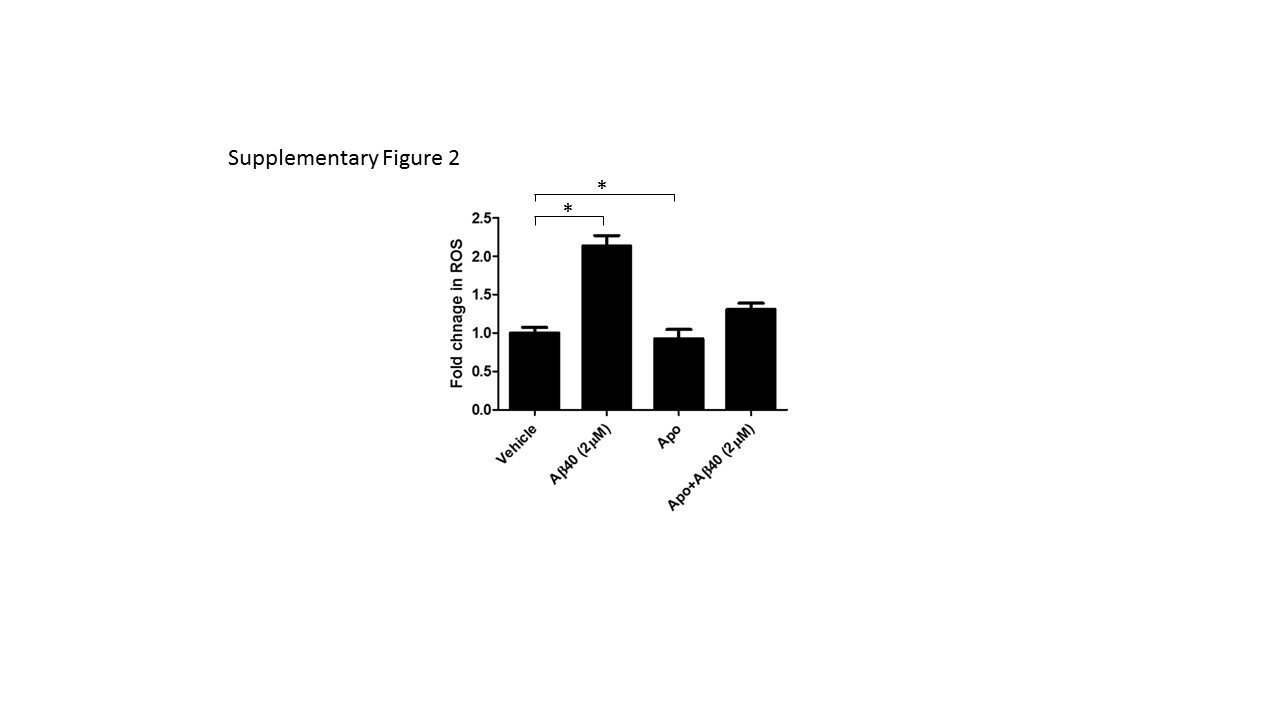

Supplement: Additional file 3: Figure S2. — Apocynin does not impact baseline ROS production in VSMC. Human VSMC were loaded with Mitotracker Red CM-H2XRos (5 μM) and treated with Aβ1-40. In some cases, cells were co-treated with the NADPH oxidase inhibitor apocynin (Apo; 10 μM). Fluorescence was measured after 30 minutes. Results are representative of 3 independent experiments performed in triplicate. *p < 0.05 vs. vehicle-treated control. #p < 0.05 vs. comparison group. (JPEG 31 kb) [file 13024_2016_73_MOESM3_ESM.jpeg]

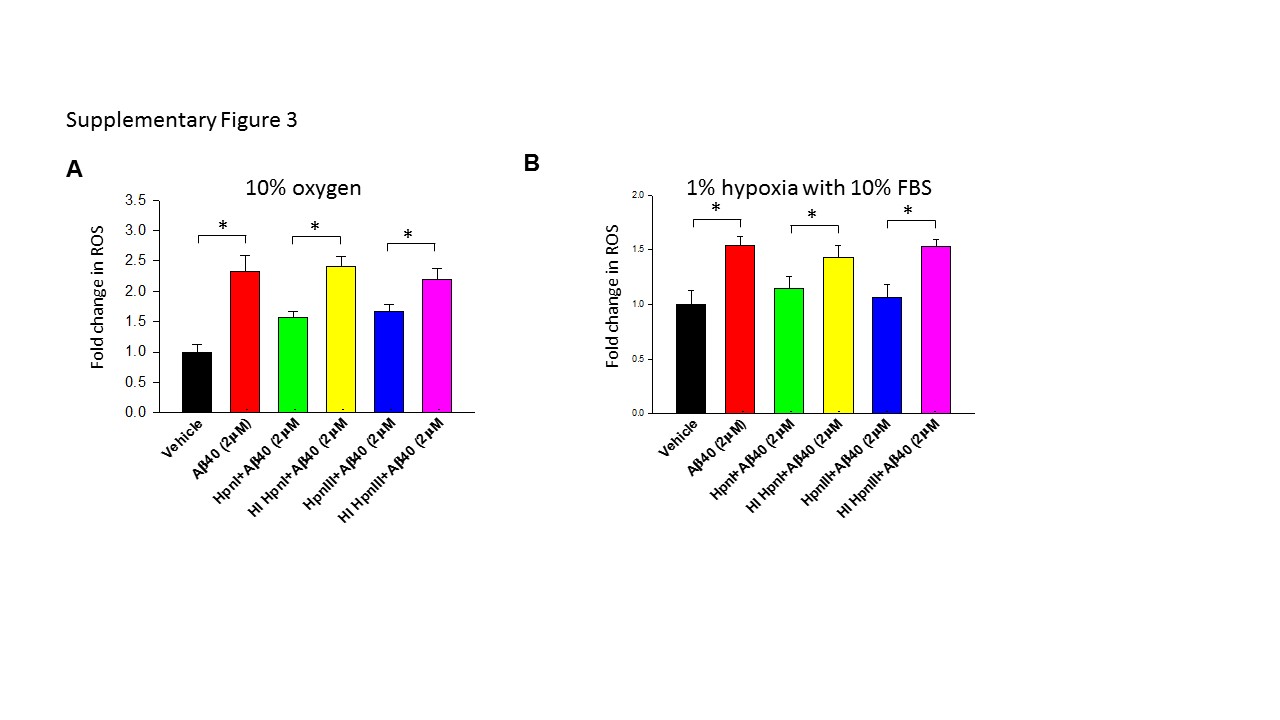

Supplement: Additional file 4: Figure S3. — HSPG mitigates Aβ1-40-induced mitochondrial and cytosolic ROS production in VSMC under physiological oxygen concentration. To determine if differing levels oxygen impact ROS production in Aβ1-40 treated VSMC, cells were kept in 10 % oxygen (Panel A) or 1 % oxygen (conditions that are considered hypoxic; Panel B) in cell culture incubator with % 5 CO2. Primary human cerebral VSMC were pre-treated with heparin (15 U/mL), heparinase I (HpnI; 5 Sigma U/mL), or heparinase III (HpnIII; 2 Sigma U/mL) for 2 h, washed, loaded with Mitotracker Red CM-H2XRos, washed, and treated with Aβ1-40. In some cases, cells were pre-treated with heat-inactivated (HI) enzyme. Fluorescence was measured after 30 minutes. Results are representative of 3 independent experiments performed in triplicate. *p < 0.05 vs. vehicle-treated control. #p < 0.05 vs. comparison group. (JPEG 70 kb) [file 13024_2016_73_MOESM4_ESM.jpeg]

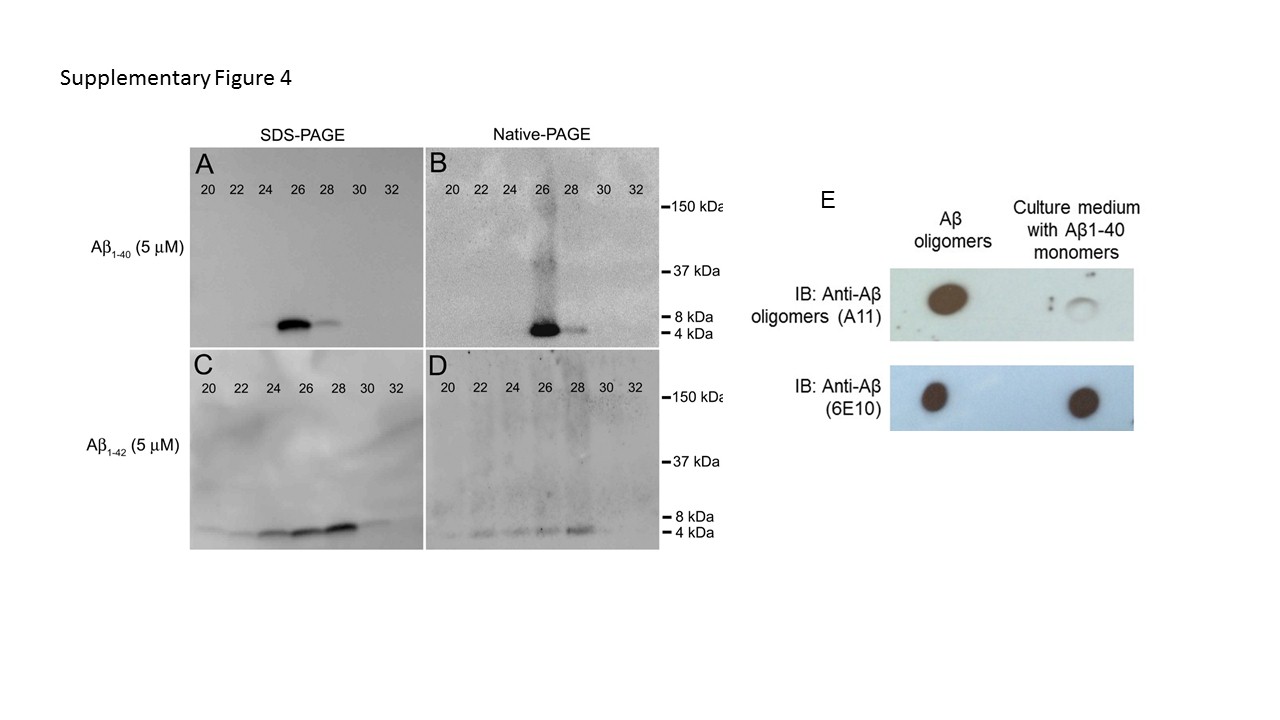

Supplement: Additional file 5: Figure S4. — Aβ1-40 and Aβ1-42 remain mostly in soluble, monomeric form after 30 minute incubation in L-15 media. Conditioned media from Aβ1-40- (panels A, B) and Aβ1-42-treated VSMC (5 μM) was loaded onto a size exclusion column and fractionated via fast performance liquid chromatography (FPLC). Aβ-containing fractions were identified using enzyme-linked immunosorbent assays (ELISA) and higher-order Aβ species were separated from monomer (4 kDa) by SDS- (panels A, C) and native- (panels B, D) polyacrylamide gel electrophoresis (PAGE) with Western blotting. Aβ1-40 and Aβ1-42 peptides were detected using the anti-Human Aβ mouse 82E1 monoclonal antibody (1 μg/mL). To specifically differentiate monomers vs. oligomers in the media, we performed dot blot using oligomer-specific anti- Aβ antibody (A11; Panel E). (JPEG 75 kb) [file 13024_2016_73_MOESM5_ESM.jpeg]

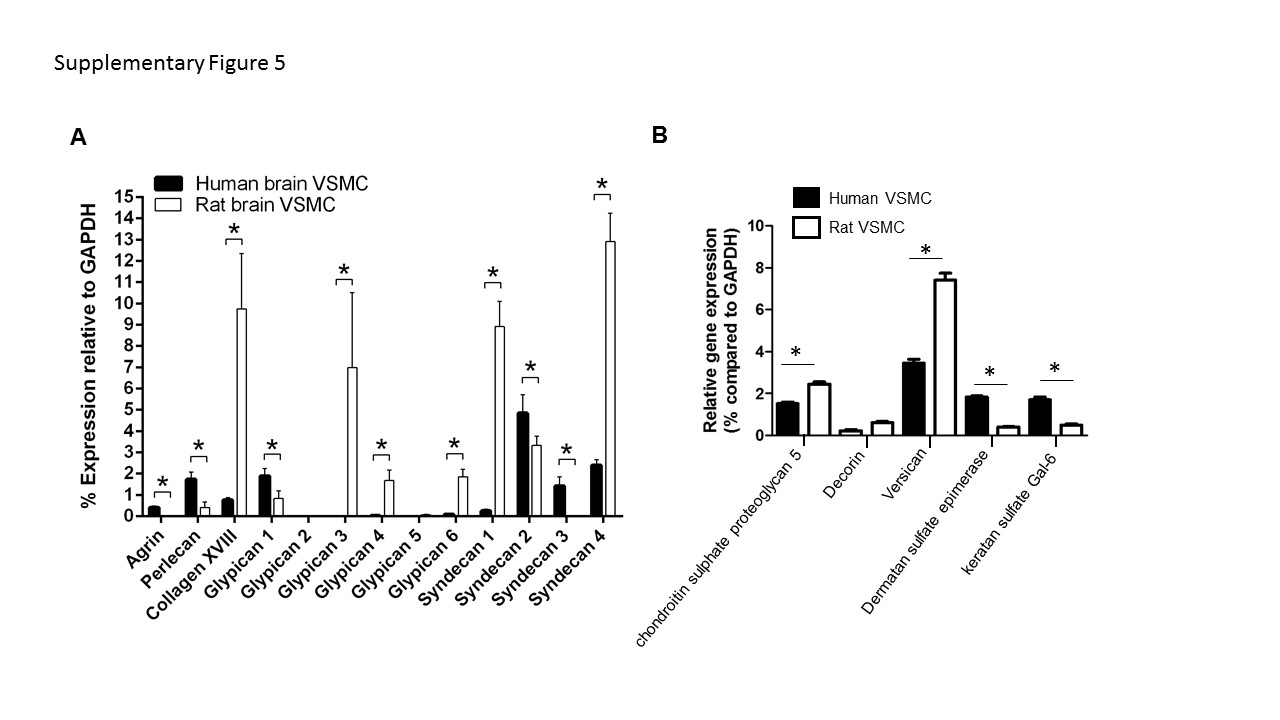

Supplement: Additional file 6: Figure S5. — HSPG subtype mRNA is present in primary human cerebral and transformed rat cerebral VSMC. HSPG subtype mRNA was harvested from primary human cerebral as well as transformed rat cerebral VSMC and measured using quantitative polymerase chain reaction (qPCR). Extracellular matrix HSPG include agrin, perlecan, and collagen XVIII; cell surface HSPG include glypicans 1-6 and syndecans 1-4 (Panel A). In addition we also performed qPCR for chondroitin sulphate, dermatan sulphate and keratan sulfate (Panel B). *p < 0.05 vs. comparison group. (JPEG 90 kb) [file 13024_2016_73_MOESM6_ESM.jpeg]

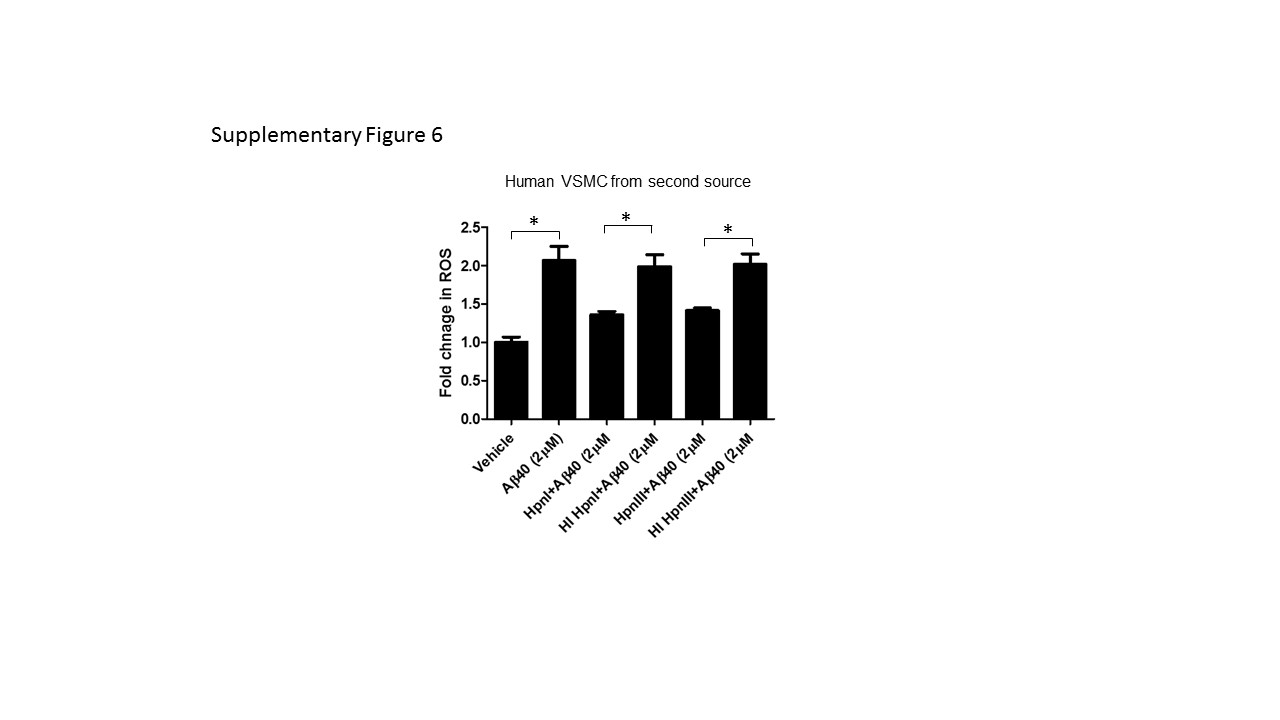

Supplement: Additional file 7: Figure S6. — Human VSMC cells from second source conform that HSPG mitigates Aβ1-40-induced mitochondrial and cytosolic ROS production in VSMC. Primary human cerebral VSMC from other source (Cell Biologics, Chicago, IL) were pre-treated with heparin (15 U/mL), heparinase I (HpnI; 5 Sigma U/mL), or heparinase III (HpnIII; 2 Sigma U/mL) for 2 h, washed, loaded with Mitotracker Red CM-H2XRos (MTR; 5 μM), washed, and treated with Aβ1-40. In some cases, cells were pre-treated with heat-inactivated (HI) enzyme. Fluorescence was measured after 30 minutes. Results are representative of 3 independent experiments performed in triplicate. *p < 0.05 vs. vehicle-treated control. #p < 0.05 vs. comparison group. (JPEG 42 kb) [file 13024_2016_73_MOESM7_ESM.jpeg]

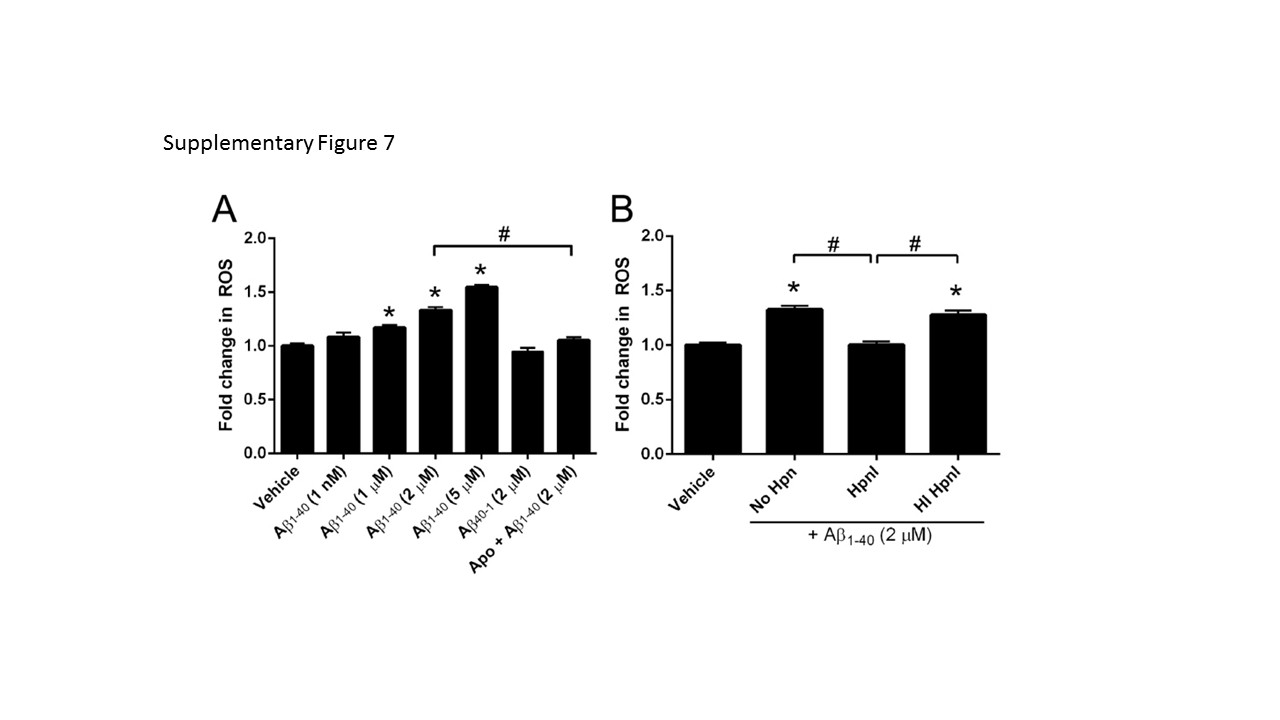

Supplement: Additional file 8: Figure S7. — Transformed rat cerebral VSMC behave similarly to human cerebral VSMC with respect to Aβ1-40-induced ROS production and mitigation of Aβ1-40-induced oxidative stress by pharmacological knockdown of HSPG with heparinase. Rat cerebral VSMC were loaded with Mitotracker Red CM-H2XRos (5 μM) and treated with varying concentrations of Aβ1-40 or a scrambled control peptide (Aβ40-1; panel A). In some experiments, cells were pre-treated with heparinase (or heat-inactivated enzyme) for 2 h, washed, and then treated with Aβ1-40 (2 μM) (panel B). Fluorescence was measured after 30 minutes. Results are representative of 3 independent experiments performed in triplicate. Apo = apocynin. Hpn = heparinase. HI = heat inactivated. *p < 0.05 vs. vehicle-treated control. #p < 0.05 vs. comparison group. (JPEG 56 kb) [file 13024_2016_73_MOESM8_ESM.jpeg]

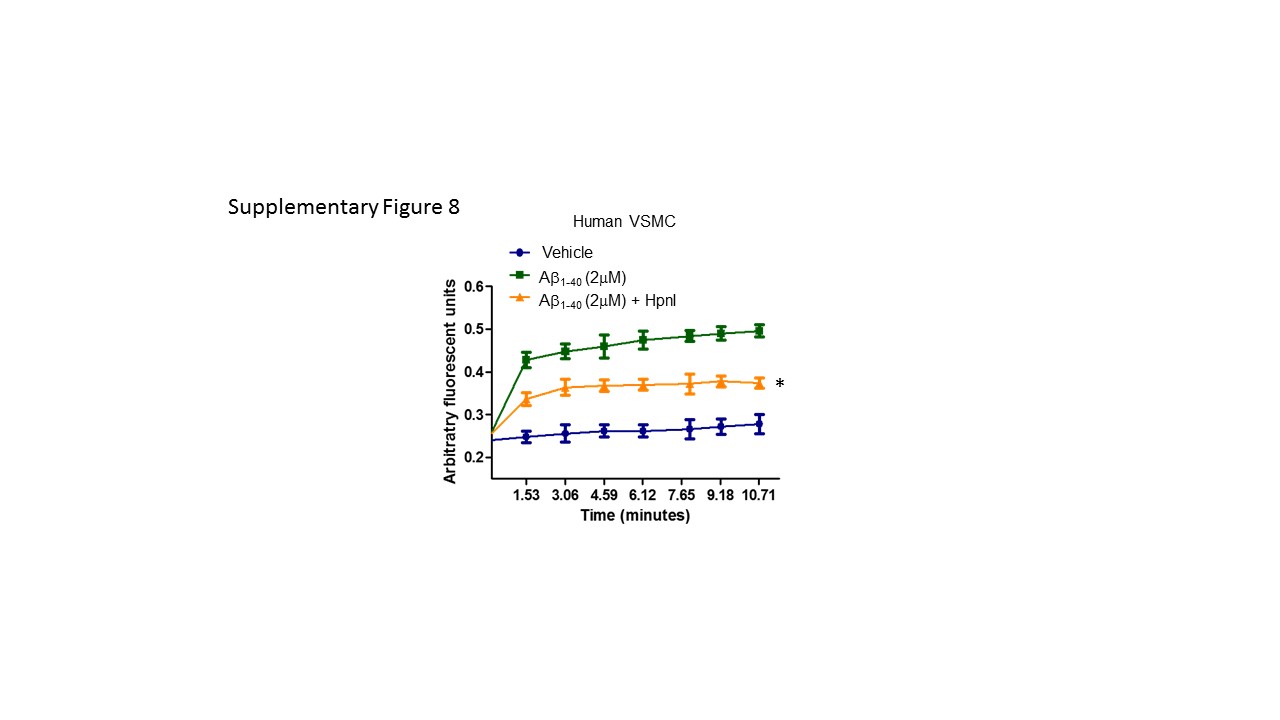

Supplement: Additional file 9: Figure S8. — Human VSMC cells also show Aβ1-40 induces HSPG-mediated Ca2+ influx. Primary human VSMC were loaded with fura II (10 μM) and treated with varying concentrations of Aβ1-40. In some experiments, cells were pre-treated with active heparinase I (HpnI; 5 Sigma U/mL), washed, loaded with fura II, and treated with Aβ1−40. Fluorescence was measured over ~10 minutes. Results are representative of 3 independent experiments performed in triplicate. *p < 0.05 vs. vehicle-treated control. (JPEG 44 kb) [file 13024_2016_73_MOESM9_ESM.jpeg]
